# Cyclization-Modulated Structure and Oxidative Behavior of Methoxy-Substituted Chalcones: Implications for Diesel-Biodiesel Additive Design

**DOI:** 10.1021/acsomega.6c05813

**Published:** 2026-09-02

**Authors:** Renata Layse G. de Paula, Vitor S. Duarte, Pollyana P. Firmino, Javier Ellena, Gábor Bognár, Pál Perjési, Júlio César de O. Ribeiro, Valter H Carvalho-Silva, Allen G. Oliver, Hamilton B. Napolitano

**Affiliations:** † Center for BioMolecular Stability of the Cerrado, 425885 State University of Goiás, Anápolis, Goiás 75132-903, Brazil; ‡ Department of Chemistry and Biochemistry, 6111 University of Notre Dame, Notre Dame, Indiana 46556, United States; § Sao Carlos Institute of Physics, University of Sao Paulo, Sao Carlos, São Paulo 05508-900, Brazil; ∥ Institute of Pharmaceutical Chemistry, University of Pécs, Pécs 7622, Hungary; ⊥ Institute of Chemistry, Federal University of Goiás, Goiânia, Goiás 74605-220, Brazil

## Abstract

The oxidative instability of diesel-biodiesel blends remains a critical challenge for fuel quality, motivating the search for new antioxidant candidates. In this study, a novel methoxy-substituted cyclic chalcone **1** was synthesized to investigate how intramolecular cyclization influences supramolecular assembly, electronic properties, and oxidative-response behavior in comparison with a structurally related open-chain analog **2**. Single-crystal X-ray diffraction revealed that cyclization induces pronounced conformational rigidity and alters the torsion angles governing intermolecular interactions in the solid state. Density functional theory calculations showed that the cyclic chalcone exhibits a larger HOMO-LUMO gap, greater chemical hardness, and lower electrophilicity, consistent with a more rigid and electronically less perturbed structure. Under oxidative stress conditions, compound **1** was less affected than **2**, indicating greater resistance to oxidative transformation. Rancimat experiments showed that the addition of compound **1** to diesel-biodiesel blends resulted in a slight increase in the induction period during storage, consistent with its greater oxidative persistence, although the stabilization effect remained modest under the evaluated conditions. Machine-learning predictions of hydroxyl-radical reaction rate coefficients revealed distinct oxidative-response profiles for the cyclic and open-chain systems, with predicted rate constants exceeding those of representative diesel-biodiesel components and commercial antioxidants such as butylhydroxytoluene, tert-butylhydroquinone, and butylhydroxyanisole. The results demonstrate that cyclization modulates the balance between molecular reactivity and oxidative persistence, providing new insights into structure-property relationships relevant to the rational design of future antioxidant additives for diesel-biodiesel blends.

## Graphical abstract

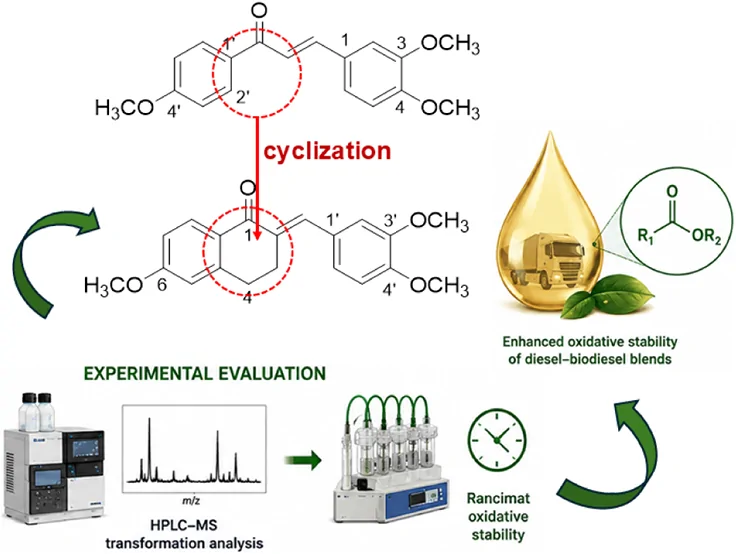

## 1 Introduction

The growing demand for clean and sustainable energy has increased interest in alternative sources, such as biofuels.^1–4^ Compared to conventional fossil fuels, biodiesel is a biofuel that offers environmental advantages, such as reduced greenhouse gas (GHG) and air pollutant emissions, high biodegradability, lower toxicity, and the use of renewable feedstocks.^5,6^ There are several international policies that promote the addition of biodiesel to petroleum diesel (blends), including blending mandates, fuel quality standards, and decarbonization targets established by regulations such as the U.S. Renewable Fuel Standard (RFS) and the European Union’s Renewable Energy Directive (RED).^7–10^ These frameworks reinforce the role of biofuels in the global energy transition, contributing to the achievement of the United Nations Sustainable Development Goals (SDGs), particularly *affordable* and *clean energy* and *climate action*. In parallel, significant advances have been achieved in biodiesel production through the development of highly efficient catalytic and photocatalytic systems. Recent studies have demonstrated that rational catalyst design, defect engineering, and microenvironment modulation can substantially improve reaction efficiency and selectivity, highlighting the rapid evolution of biodiesel technologies and reinforcing the strategic importance of complementary approaches aimed at improving fuel quality and long-term storage stability.^11–13^

Despite these remarkable advances in biodiesel production technologies, biodiesel remains particularly susceptible to oxidative degradation because of its high content of unsaturated fatty acid methyl esters.^14^ As a result, blending biodiesel with petroleum diesel tends to reduce the oxidative stability of the final fuel, making it more prone to the formation of peroxides, gums, and other degradation products that can impair engine performance and durability.^15^ This vulnerability to oxidation remains one of the main obstacles to large-scale biodiesel consolidation, as it limits storage stability, shelf life, and compatibility under real operating conditions.^16^ To mitigate these effects, the development of efficient antioxidant additives remains a key challenge in fuel chemistry. Recent reviews have reinforced that oxidative stabilization remains a major technological bottleneck for biodiesel-containing fuels and that antioxidant efficiency depends not only on radical-quenching ability, but also on factors such as molecular persistence, solubility, and compatibility with the fuel matrix.^17–19^

Beyond improvements in biodiesel production, current research has increasingly focused on the development of predictive methodologies capable of accelerating fuel optimization. Recent studies have demonstrated that the integration of machine learning with experimental biodiesel systems provides a powerful approach for predicting engine performance and optimizing fuel properties.^2,20,21^ Chalcones and their derivatives have emerged as promising antioxidant candidates due to their *π*-conjugated systems, electron-donating substituents, and structural adaptability, which enable a wide range of redox and radical-scavenging activities.^22–25^ Methoxy-substituted chalcones exhibit advantageous characteristics for antioxidant applications, as the methoxy groups can favor interactions with radical species.^26^ Furthermore, structural modifications, such as intramolecular cyclization, can significantly influence molecular stability, packing efficiency, and electronic distribution parameters directly related to antioxidant performance with contributions that are directly relevant to oxidative behavior.^27^ However, the relationship between the structural rigidity of a chalcone, its molecular reactivity, and its effectiveness as a radical scavenger in nonpolar media, such as diesel and biodiesel, remains poorly explored. In particular, it remains unclear whether cyclization favors antioxidant-related performance by enhancing radical reactivity, by increasing resistance to oxidative transformation, or by modulating both features concurrently.

In this context, a novel methoxy-substituted cyclic chalcone-(2*E*)-2-[(3′,4′-dimethoxyphenyl)­methylidene]-6-methoxy-3,4-dihydronaphthalen-1­(2*H*)-one (**1**), was synthesized and analyzed to investigate how cyclization near the aromatic nucleus can influence physicochemical and antioxidant properties. Additionally, a structurally similar open-chain chalcone, retrieved from the Cambridge Crystallographic Data Centre (CCDC) under code 2080859 (**2**),^28^ was selected to allow comparison of the structural differences induced by the incorporation of a fused six-membered ring to the chalcone moiety (Figure 1a). This pair of compounds offers a useful model for isolating the structural consequences of cyclization while minimizing unrelated substitution effects.

**Figure 1:**
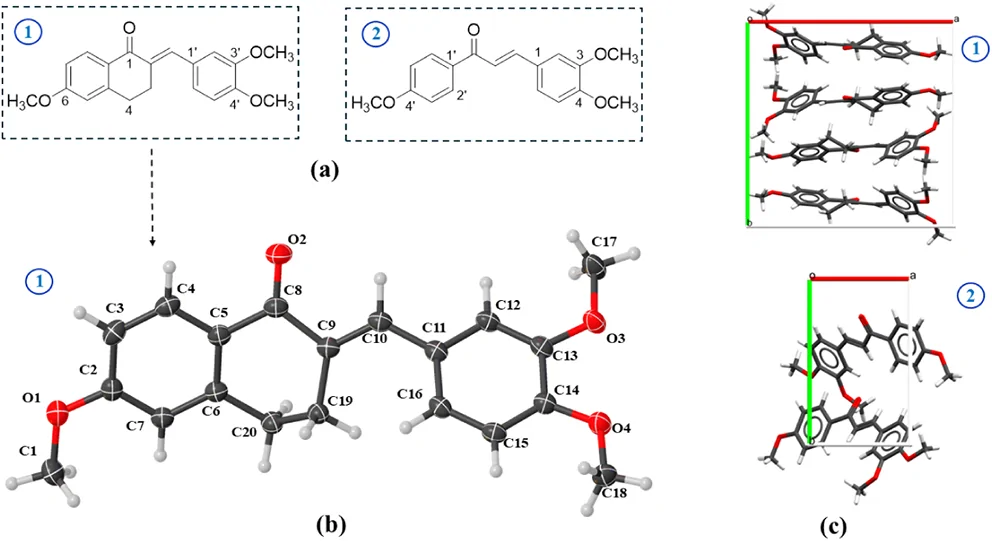
**(a)** Structural formulas of the **1** cyclic chalcone and **2** a similar open-chain chalcone obtained from the CCDC database.^28^ **(b)** ORTEP type view of the asymmetric unit of **1** with displacement ellipsoids at the 50% probability level, and hydrogen atoms are shown as spheres of arbitrary radius. **(c)** Molecular packing in the unit cell for compounds **1** and **2**.

Recent studies from our research group have systematically investigated the reactivity of (*E*)-2-benzylidenecyclanone derivatives toward biological nucleophiles, particularly thiols, using thia-Michael addition as a model reaction.^29,30^ Experimental results revealed that electrophilic reactivity is strongly dependent on the nature of the cyclized structure, rather than following a simple trend based solely on ring size. Notably, 4-chromanone derivatives exhibited unexpectedly higher reactivity than the structurally related tetralone analogs, highlighting the influence of the oxygen atom within the ring on the electronic distribution and activation of the *β*-carbon in the *α,β*-unsaturated system.^29^ In parallel, comparative analyses involving indanone, tetralone, and benzosuberone derivatives demonstrated that variations in ring size and conjugation length lead to significant differences in reactivity toward thiol nucleophiles, which were associated with changes in steric accessibility and *π*-electron delocalization.^30^ These findings indicate that both electronic effects and conformational constraints imposed by cyclization play a decisive role in modulating the reactivity of cyclic chalcone analogs. In addition, we have shown that methoxy-substituted chalcones can act as promising antioxidant additives in diesel-biodiesel blends, both in terms of oxidative-stability enhancement and structure-based interpretation, thereby providing a direct practical background for the present work.^28,31^

Additionally, in the literature, there are recent studies demonstrating that chalcone derivatives exhibit significant antioxidant activity, which is strongly influenced by structural and electronic factors such as hydroxyl substitution patterns, *π*-electron delocalization, and molecular conformational flexibility.^22^ Investigations have shown that radical-scavenging processes in chalcone systems may proceed through hydrogen atom transfer (HAT), single-electron transfer (SET), proton-coupled electron transfer (PCET), and the Sequential Proton Loss Electron Transfer (SPLET) mechanism, highlighting the importance of both thermodynamic and electronic descriptors in governing antioxidant behavior.^22,32,33^ Recent reviews further indicate that structural modification of the chalcone scaffold, including cyclization or conformational restriction, can significantly influence electron distribution and molecular reactivity, thereby modulating antioxidant performance.^22,34,35^ In particular, cyclic or structurally constrained chalcone derivatives have been reported to alter *π*-conjugation and steric accessibility, which may affect radical-scavenging efficiency and stability of radical intermediates.^22,36^ Earlier studies with (*E*)-2-(4′-methoxybenzylidene)-1-tetralone (an analog of **1**) showed the compound to possess a mild antioxidant (hydroxyl radical scavenging) effect in the Fenton-reaction initiated deoxyribose degradation test. Similar to its open-chain analog, the compounds showed the highest activity at the 60-minute timepoint.^37^

However, most reported studies on chalcone and cyclic chalcone derivatives remain focused on biological or model radical assays, with limited attention given to their oxidative persistence or stability under conditions relevant to complex chemical environments.^38^ In this context, the present study extends previous findings by integrating literature-based insights with computational predictions, reaction pathway simulations, and experimental oxidation tests, enabling a more comprehensive evaluation of both radical-scavenging reactivity and structural resistance to oxidative transformation in diesel-biodiesel fuel systems.

Although previous studies have shown that cyclization modulates the electrophilic reactivity of benzylidenecyclanone derivatives toward nucleophiles, its effect on radical-scavenging behavior and on the oxidative stability of diesel-biodiesel systems remains unclear. In this study, we compare the cyclic chalcone **1** with a structurally related open-chain analog **2** to test whether intramolecular cyclization alters molecular geometry, supramolecular organization, and electronic structure in ways that affect antioxidant-related behavior. More specifically, we hypothesize that cyclization modifies the balance between molecular reactivity and oxidative persistence by increasing conformational rigidity and altering electronic descriptors associated with radical-mediated transformation. Here, we aimed to: (i) determine how cyclization influences molecular conformation, crystal packing, and intermolecular contacts; (ii) evaluate its impact on electronic descriptors and predicted reactivity toward hydroxyl radicals; and (iii) assess whether these changes are associated with measurable improvements in the oxidative stability of S10 B14 diesel during storage, in comparison with the neat fuel and a commercial additive. By integrating single-crystal X-ray diffraction, DFT calculations, Fenton-based reactivity assessment, machine-learning predictions, and Rancimat experiments, this work seeks to establish a clearer structure-activity relationship for cyclic chalcones as prospective antioxidant candidates for diesel-biodiesel blends. Thus, rather than assuming that cyclization simply enhances antioxidant performance, this study examines how cyclization modulates the relationship between structure, electronic response, oxidative transformation, and fuel-stabilization behavior.

## 2 Experimental and Computational Procedures

### 2.1 Synthesis and Structural Characterization

(*E*)-2-(3,4-Dimethoxybenzylidene)-6-methoxy-3,4-dihydronaphthalen-1­(2H)-one (**1**). 5.0 mL of ethanol solution of 10 mmol (1.8 g) of 3,4-dimethoxybenzaldehyde was added to a mixture of 5.0 mL ethanol solution of 10 mmol (1.7 g) of 6-methoxy-1-tetralon and 5.0 mL of ethanol solution of 20 mmol (1.1 g) KOH, and the mixture was stirred at room temperature for 24 hours. After completion of the reaction, the mixture was diluted with 15 mL of distilled water, and the resulting precipitate was collected and dissolved in 100 mL of chloroform. The chloroform solution was washed free of the base with distilled water, the organic phase was separated, dried on anhydrous Na_2_SO_4_, and evaporated. The obtained solid was crystallized from ethanol to give pale yellow crystals. The purity of the compound was tested by TLC (Merck silica gel 60 F_254_ precoated alumina plates; toluene:ethanol = 10:1 (v/v) mixture). Yield: 70.1%. M.p. 110–112 °C (decomp.). The structure of the obtained compound was characterized by IR, NMR, and MS methods. IR (IMPACT 400 (Nicolet); KBr, ν (cm^–1^)): 3002, 2987, 2960, 2946, 2931, 2908, 2834, 1660, 1606, 1591, 1513, 1465, 1447, 1326, 1255, 1164, 1145, 1136, 1028, 968, 917, 906, 863, 850, 831, 821, 765, 621, 562. ^1^H NMR (Bruker Avance III 500; 500 MHz, CDCl_3_, δH (ppm)) 8.13 (1H, d, J= 8.7 Hz), 7.81 (1H, brs), 7.08 (1H, dd, J = 8.3 Hz, 1.3 Hz), 7.00 (1H, d, J = 1.3 Hz), 6.93 (1H, d, J= 8.3 Hz), 6.89 (1H, dd, J = 8.7 Hz, 2.3 Hz), 6.73 (1H, d, J = 2.3 Hz), 3.94 (3H, s), 3.93 (3H, s), 3.89 (3H, s), 3.16 (2H, dt J = 6.4 Hz, 1.3 Hz), 2.94 (2H, t, J= 6.4 Hz). ^13^C NMR (Bruker Avance III 500; 125.7 MHz, CDCl_3_, δC (ppm)): 186.64, 163.52, 149.53, 148.82, 145.52, 136.12, 134.04, 130.71, 128.93, 127.22, 123.15, 113.37, 113.25, 112.28, 111.05, 55.98, 55.97, 55.43, 29.25, 27.32. LC-MS (HPLC Ultimate 3000 coupled with Q Exactive Focus Mass Spectrometer) [M+1] ^+^ 325.1430.

Crystal structure analysis of **1** was conducted using X-ray diffraction data collected on a Rigaku XtaLAB Synergy-S diffractometer, equipped with a HyPix-6000HE detector and using a CuKα radiation (λ = 1.54184 Å) microfocus sealed X-ray source. The crystal was kept steady at T = 100,0(2) K during data collection. Data collection, data reduction, cell refinement, and gaussian absorption correction were performed using CrysAlisPro software.^39^ The structure was solved with the SHELXT structure solution program using the Intrinsic Phasing method^40^ and Olex2 as the graphical interface.^41^ The model was refined with ShelXL-2019/2 using the Least Squares minimization method,^42^ with the non-hydrogen atoms refined considering anisotropic displacement parameters. The hydrogen atoms were refined at idealized positions, using the riding model. Crystallographic data, refinement parameters, and additional information can be found in (Supporting Information Tables S1–S8). The crystallographic data for **1** have been deposited in the Cambridge Crystallographic Data Centre (CCDC)^43^ under the deposition number 2550843. Based on the structural data obtained from single-crystal X-ray diffraction, the solid-state analysis was carried out using the molecular geometries, through crystal packing, intermolecular interactions, and supramolecular arrangement, using the Mercury software,^44^ as well as through electron density with Hirshfeld surface (HS) analysis^45,46^ performed with CrystalExplorer.^47^ Furthermore, the intermolecular contacts were quantified using the corresponding 2D fingerprint plots.^48^ Hirshfeld surfaces are constructed from normalized contact distances (d_norm_), defined by the distance from the surface to the nearest atom inside the molecule (d_e_) and to the nearest atom in a neighboring molecule (d_i_),^45^ while the 2D fingerprint plots provide graphical representations of the d_e_ vs. d_i_ distributions.^48^

### 2.2 Oxidation Stability Test

The accelerated oxidative stability test (Rancimat test) was performed for **1** added to a diesel-biodiesel blend containing 14% biodiesel and 86% petroleum diesel (commercial Diesel S10 B14). The fuel was obtained from a commercial gas station in Brazil, where the 14% biodiesel blend (B14) has been mandatory since March 2024 (CNPE Resolution No. 8/2023; EPE report).^49^ The Rancimat analysis was carried out in accordance with the requirements established in the EN 15751 standard.^50^ This methodology applies to fatty acid methyl esters (FAME) used as biofuels either in pure form or blended with mineral diesel at concentrations of at least 2% (v/v). In the Rancimat test, the fuel is heated at 110 °C and exposed to a purified air flow that promotes oxidation, leading to the formation and subsequent decomposition of peroxides into volatile acids. These volatiles are transported to a sealed vessel containing deionized water, where the rapid increase in conductivity, measured by an electrode, marks the end of the induction period. Oxidation stability is expressed as the length of the Induction Period (IP) in hours. Although there is no legislation establishing a minimum oxidation stability limit at 110 °C for diesel-biodiesel blends sold in Brazil, the ANP Resolution no. 920/2023 specifies that commercial biodiesel must exhibit a minimum oxidation stability of 13 h at 110 °C.

Analyses were performed using an 893 Professional Rancimat (Metrohm) instrument in accordance with EN 15751:2014.^50^ The S10 B14 diesel samples were initially analyzed on Day 0 and subsequently after 50 and 100 days of storage in 250 mL opaque polyethylene terephthalate (PET) containers. Each bottle was filled to approximately 30% of its total volume, allowing for an air headspace, enabling contact with oxygen, and thereby promoting oxidative processes under storage conditions. The S10 B14 diesel was blended with **1** at a concentration of 1000 ppm and analyzed after the same storage intervals (50 and 100 days). A commercial antioxidant additive (CA) was also evaluated under identical conditions for comparison. All samples were maintained at room temperature and protected from direct. Measurements were performed in triplicate (T1–T3), and the results were expressed as mean induction period values in hours. The raw triplicate data and the summarized mean ± SD and mean ± SE values are reported in Tables S14a–S14c of the Supplementary Information.

### 2.3 Fenton Oxidation Test

To 400 μL of sulfuric acid (pH 3.0), 250 μL of 30 mM iron­(II) sulfate, and 100 μL of 1.0 mg/mL methanolic chalcone (**1** or **2**) solution were added with vortexing. Then, 250 μL of 10 mM hydrogen peroxide solution was added, and the mixture was incubated in a 37 °C shaking water bath for 60 minutes. “Blank” samples contained distilled water instead of hydrogen peroxide. Three parallel samples were made for each compound. The 60-minute incubates were analyzed by HPLC-UV method. The measurements were performed on an Agilent 1100 HPLC system coupled with a UV-VIS detector. The wavelength was set at 254 nm. The components were separated using a reversed-phase chromatographic system. A PerfectSil 120 C8 (100 mm × 4.6 mm, particle size 5 μm) column (Agilent Technologies, Waldbronn, Germany) was used to separate the components. The injection volume was 10 μL. Data was recorded and evaluated using Agilent ChemStation (B.03.01). Isocratic elution was performed at a flow rate of 1.0 ml/min, using acetonitrile:water 50:50 v/v mixture as the eluent. The reactivity of the compounds to the free radicals was determined by comparing the peak area (AUC) of the chalcone investigated in the reference and Fenton samples at 60 minutes. The Fenton assay was not intended to reproduce the chemical environment of diesel-biodiesel blends. Instead, it was employed as a standardized oxidative-stress model to compare the relative susceptibility of the two chalcone structures toward hydroxyl-radical attack. Consequently, the results should be interpreted as mechanistic evidence of oxidative-response behavior rather than direct evidence of antioxidant performance in fuel media.

### 2.4 HPLC-MS Analysis

HPLC-HESI-MS analyses were performed on an UltiMate 3000 liquid chromatograph (Dionex, Sunnyvale, CA, USA) coupled with a Thermo Q Exactive Focus quadrupole-Orbitrap hybrid mass spectrometer (Thermo Fisher Scientific, Waltham, MA, USA). The scan monitored m/z values ranging from 100 to 1300 Da. Data was acquired using Q Exactive Focus 2.11 and Xcalibur 4.2 software (Thermo Fisher Scientific). Analysis of compounds and adducts was performed in HESI positive and negative ionization modes with the following parameters: spray voltage, 3500 V; vaporizer temperature, 300 °C; capillary temperature, 350 °C; spray and auxiliary gas flows, 30 and 10 arbitrary units, respectively; resolution, 35,000 at 200 m/z.

HPLC separation was performed on an Accucore RP-MS column (150 mm × 2.1 mm, particle size 2.6 μm), and an Accucore C18 guard column (5 mm × 2.1 mm, particle size 2.6 μm) was also used. The injection volume was 5 μL; the flow rate was set to 0.4 mL/min. Data analysis and evaluations were performed using Xcalibur 4.2 software. A binary gradient of eluents, consisting of mobile phases A and B, was used.

The gradient parameters were (A) water and 0.1% formic acid and (B) methanol and 0.1% formic acid. The gradient elution was as follows: isocratic elution for 1 min to 10% eluent B, continued by a linear gradient to 95% in 13 min, followed by an isocratic plateau for 3 min. Finally, the column was equilibrated to 10% in 0.1 min and continued isocratically for 2.9 min. The sampler was at room temperature, and the column oven was at 40 °C.

### 2.5 Computational and Machine Learning Methods

The molecular and electronic structures of **1** were analyzed using DFT^51^ implemented in the Gaussian 09 software package.^52^ The compound was analyzed in three phases: optimized in the gas-phase (I), with implicit solvent using *n*-hexadecane (*via* its dielectric constant) (II), as well as through single-point energy calculations (III) in the solid-state (X-ray geometry), in which the molecular geometry is not optimized, and only the energy of the fixed structure is obtained. All calculations were performed at the M06-2X/6–311++G­(d,p) theory level,^53,54^ which provides satisfactory results by adequately accounting for electronic correlation and noncovalent interactions.^53,55^ Gas-phase optimization offers a description of the isolated molecular geometry, free from intermolecular effects, whereas optimization using the dielectric constant of *n*-hexadecane yields a structure more representative of the molecule in a diesel-like environment. For the implicit optimization with *n*-hexadecane, the Conductor-like Polarizable Continuum Model (CPCM) was used, with a dielectric constant (ε) of 2.0402.^56,57^ From the wave function generated in these calculations, the frontier molecular orbitals (FMOs) were determined: the highest occupied molecular orbital (HOMO) and the lowest unoccupied molecular orbital (LUMO).^58^ From these parameters it is possible to determine some chemical reactivity descriptors, such as, the energy difference between the orbitals HOMO and LUMO (*E*_GAP_), the ionization potential (*I*), the electron affinity (A), the chemical potential (*μ*), the electronegativity (*χ*), the chemical hardness (*η*), and the electrophilicity index (*ω*). The Molecular Electrostatic Potential (MEP) maps were generated to visualize charge distribution and identify reactive sites. The electrostatic potential was calculated based on the electron density obtained at the same level of theory used for the optimized structures.^59^ Same calculations were also performed for **2** to provide a comparative benchmark.

To complement the DFT analysis, oxidative reactivity toward hydroxyl radicals was estimated using the *py*SiRC platform.^60^ This platform allows prediction of the reaction rate constant (*k*_OH_), which is the rate at which a compound reacts with hydroxyl radicals (^•^OH).^60–62^ These radicals (^•^OH) are highly reactive species that are considered responsible for initiating and propagating oxidation in fuels, such as biodiesel.^63–65^ The *k*_OH_ values should be interpreted as comparative reactivity proxies toward ^•^OH, not as direct measures of antioxidant efficiency in diesel–biodiesel blend. The *k*_OH_ predictions were generated using the Molecular ACCess System keys—MACCS fingerprints^66,67^ combined with the trained model under the Extreme Gradient Boosting—XGBoost algorithm.^68^ MACCS fingerprints are 166-bit 2D structure fingerprints commonly used to measure molecular similarity.^69^ At the same time, the XGBoost algorithm employs parallelized gradient-boosted decision trees, offering high speed and accuracy in solving a wide range of data science problems, and it has been extensively adopted in recent literature.^70–73^ The platform also calculates the applicability domain (AD%), which reflects the similarity between the compounds being analyzed and those in the training set, thereby enhancing the predictive model’s reliability.^60^ The *k*_OH_ prediction can be performed for any molecule as long as its structural composition is known. Diesel S10 B14 was represented as a mixture of 86% diesel, which modeled using both an average hydrocarbon surrogate and n-hexadecane, which is often adopted as a representative paraffinic component in diesel surrogate studies. Diesel is typically composed of hydrocarbons containing approximately 8 to 21 carbon atoms, mainly paraffins ranging from C_10_H_22_ to C_15_H_28_, although authors often use the average chemical formula C_12_H_23_ to represent common diesel fuel.^74^ Among these components, *n*-hexadecane (C_16_H_34_) is frequently used as a representative model compound due to its abundance and structural similarity to the long-chain paraffins present in diesel.^75,76^ Biodiesel is composed of a mixture of esters, such as methyl oleate (C_19_H_36_O_2_), methyl palmitate (C_17_H_34_O_2_), methyl linoleate (C_19_H_34_O_2_), and methyl stearate (C_19_H_38_O_2_).^77–80^

The *k*_*OH*_ parameters were calculated for **1**, the database chalcone **2**,^28^ the major diesel components (the average molecule and *n*-hexadecane), and the biodiesel components (methyl oleate, methyl linoleate, methyl palmitate, and methyl stearate). They were compared with the parameters of commercial additives (tert-butylhydroquinone (TBHQ), butylhydroxytoluene (BHT), butylhydroxyanisole (BHA), gallic acid (GA), propyl gallate (PG), and pyrogallol (PY).^18^ The analyzed molecules were uploaded to the platform for calculation in ASCII format containing their SMILES identifiers (Table S9). The *k*_*OH*_ prediction is particularly relevant, as an antioxidant can function as a scavenger or protective shield, preferentially reacting with free radicals (such as^•^OH) to prevent them from attacking fuel molecules (biodiesel or diesel) and triggering oxidation-mediated degradation.^60–62^ Therefore, the higher the *k*_OH_ constant of an antioxidant, the faster it will theoretically react with hydroxyl radicals, consuming them before they can cause damage to the fuel.^81,82^

Reaction pathway simulations were performed using the ASKCOS platform^83^ to predict possible products formed from the interaction between **1** and hydroxyl radicals (^•^OH), as well as water (H_2_O), under diesel-like conditions. The algorithm provides ranked product candidates and associated probabilities based on data-driven forward-prediction models. In the present work, ASKCOS was used as an exploratory tool to map plausible transformation pathways and structural trends, rather than as a source of mechanistic proof. The predicted products were analyzed in terms of structural modification, preservation or disruption of conjugation, and potential oxidation-related transformation patterns. The ranked outputs used in the discussion are reported in Tables S10–S12 of the Supplementary Information.

The Ecological Structure-Activity Relationship (ECOSAR v2.0) model was applied to assess the ecotoxicological behavior of the investigated compounds. Developed by the U.S. Environmental Protection Agency (USEPA), this tool estimates aquatic toxicity by integrating quantitative structure–activity relationship (QSAR) principles with machine-learning-based predictions, using structurally similar chemicals as reference datasets.^84,85^ For this study, three representative aquatic organisms were considered: green algae, *Daphnia*, and fish. ECOSAR generated a toxicity profile for each compound, including both acute endpoints (LC_50_ and EC_50_) and chronic endpoints (ChV). The LC_50_ corresponds to the concentration of a substance (mg L^–1^) expected to cause mortality in 50% of the exposed population, determined after 96 hours for fish and 48 hours for *Daphnia*. The *EC*_50_ value indicates the concentration (mg L^–1^) that inhibits the growth of 50% of the green algal population after 96 hours of exposure. Chronic toxicity is expressed as the ChV parameter. The criteria adopted to categorize toxicity levels based on acute and chronic endpoints are summarized in Table S13.

## 3 Results and Discussion

### 3.1 Structural and Supramolecular Analysis

Single-crystal X-ray diffraction analysis reveals that **1** crystallizes in the P2_1_^/^c monoclinic system (Z = 4), with a cell volume of 1614.68 Å^3^ (Table S1), and possesses three methoxy groups, one on aromatic ring 1 and two on aromatic ring 2, located at the position 4 of ring 1, and at the 3′ and 4′ positions on ring 2. Compound **1** contains a fused six-membered ring to aromatic ring 1, in which an ethylene chain forms the condensed ring system. This structural feature (hereafter referred to as “*cyclization*”) can impose conformational restrictions and to influence both the geometry of the conjugated system and the organization of the molecule in the solid state (Figure 1b).

The comparative analysis between **1** and **2** reveals clear structural and conformational differences. The packing in the unit cell shows that **1** crystallizes in the P2_1_/c space group with four molecules in the unit cell, whereas **2** crystallizes in P2_1_ with two molecules (Figure 1c). The different space groups account for the distinct molecular multiplicities and influence the three-dimensional arrangement and intermolecular interactions. The cyclization near the aromatic ring 1 in the chalcone **1** suggests a more regular orientation of the molecules within the crystal lattice. The overlap of **1** and **2** was performed using the aromatic ring 1 (C2, C3, C4, C5, C6, and C7) as the anchoring point, yielding an RMSD of 0.0177 Å (consistent with the rigid nature of the aromatic ring). It showed that the difference in the aromatic ring 2 was 4.54°, suggesting that, although the two molecules share a similar structure, the cyclization in **1** induces changes in both the conformational structure and the crystal packing (Figure 2a). These structural variations may influence the physicochemical properties of the molecule, such as polarity, solubility, stability, and reactivity. Compound **1** shows a torsional difference of around 54° between the same regions. However, the methylene group in the cyclized region (C2 atom) does not follow the planarity of this region, as confirmed by the dihedral angle τ­(C6-C20-C19-C9) = 56.34° (Figure 2b). Similarly, **1** exhibits a planar structure in the region of aromatic ring 1 as well as in the region involving the *α,β*-unsaturated carbonyl and the aromatic ring 2 (τ­(C8-C9-C10-C11) = 177.01°), with a torsion of approximately 19° between these two regions (Figure 2c). This alteration can be attributed to the cyclization in **1**, which introduces steric constraints and modifies the local geometry around the methylene group, preventing it from maintaining planarity with the adjacent conjugated system. These results suggest that the cyclization in **1** affects the orientation of the aromatic rings and the conjugated region, potentially influencing intermolecular interactions, crystal packing, and, possibly, molecular properties.

**Figure 2:**
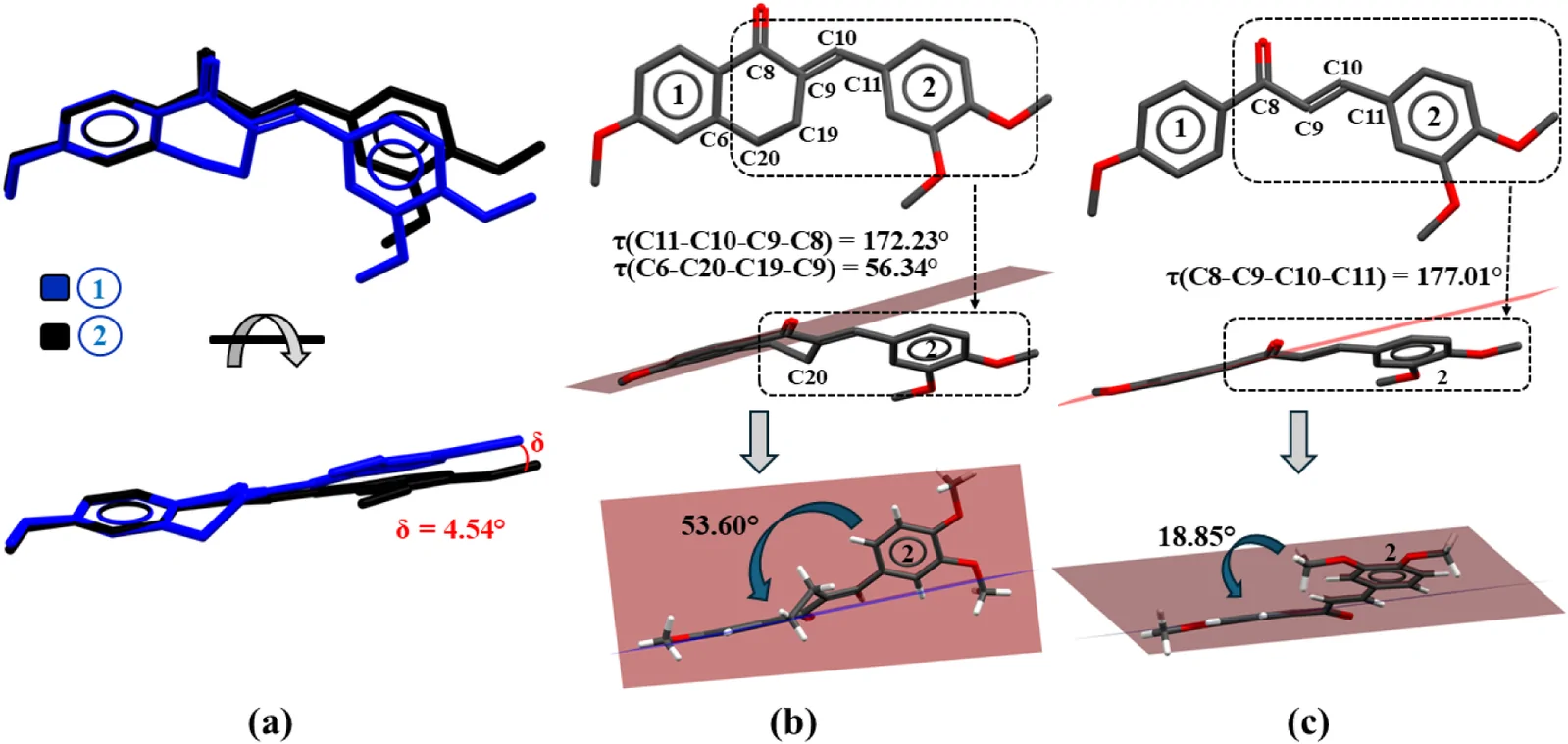
**(a)** Overlap between compounds **1** and **2**, highlighting the conformational deviation by a torsion angle δ = 4.54°. **(b)** Planarity and dihedral angles of **1** and **(c)** planarity and dihedral angles of **2**, illustrating the torsional differences between the aromatic and *α,β*-unsaturated carbonyl regions.

The crystal structure of **1** is stabilized by weak intermolecular interactions, such as C–H ··· O and C–H ··· π (Table S2). The most directional C–H ··· O interaction is C17–H17b ··· O4, characterized by a nearly linear geometry (D–H–A = 174.58(6)°) and the shortest H ··· A distance in the structure (2.430(4) Å). Other interactions such as C20–H20b ··· O2 and C1–H1b ··· O3, with H ··· A distances of 2.562(5) and 2.661(4) Å, also participate significantly in molecular packing (Figure 3a). The geometric observations are consistent with the HS analysis. The d_norm_ HS highlights regions associated with C–H ··· O interactions through intense red spots, corresponding to contacts shorter than the sum of the van der Waals radii (Figure 3b). The intermolecular contacts were further quantified using 2D fingerprint plots derived from the HS. The pseudo-mirrored spikes observed at 1.0 – 1.2 (d_e_, d_i_) indicate the presence of H···H contacts, which account for 47.8% of all interactions in the compound **1**. This high percentage of contacts involving hydrogen atoms is expected for organic molecules. Peaks in the 1.2–1.4 Å (d_e_ and d_i_) reveal the contribution of C–H ··· O interactions, corresponding to 23.3%. Contacts involving *π*-systems, particularly C–H ··· *π* interactions, are also evident, contributing 26.9% to **1**. These interactions

**Figure 3:**
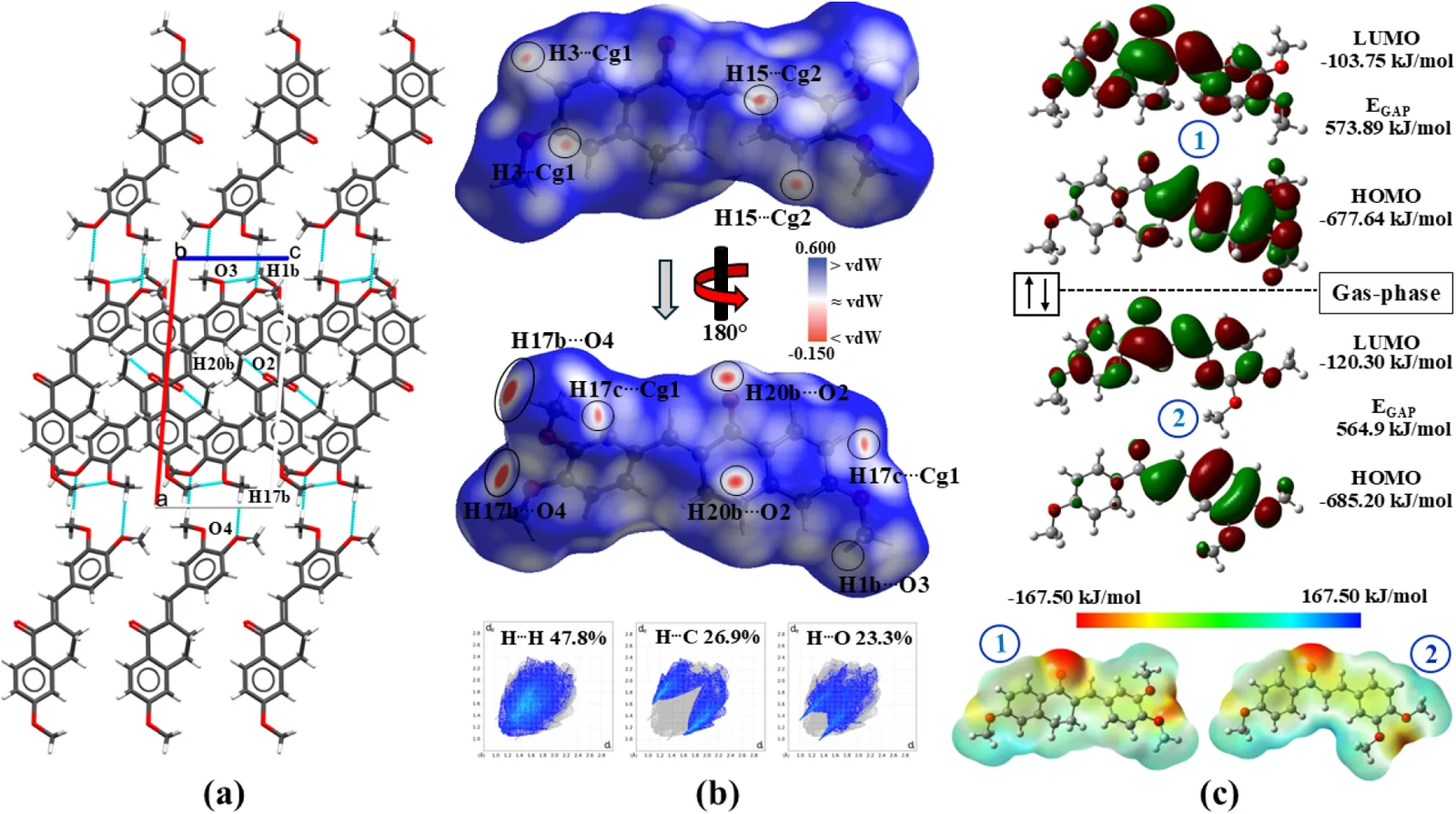
**(a)** Intermolecular interactions represented in the unit cell along the *a*-axis and **(b)** HS d_norm_ and 2D fingerprint plots indicating the interactions for **1**. **(c)** The FMO orbitals and MEP map surfaces for **1** and **2** in the gas-phase. Complete FMO orbitals and MEP map surfaces are shown in (Supporting Information Figures S1 and S2).

### 3.2 Oxidation Stability Analysis

The oxidative stability of S10 B14 diesel was evaluated over storage time using the Rancimat method, and the results show a progressive decline in induction period (IP), consistent with the expected degradation of diesel-biodiesel blends (Figure 4). Although the Rancimat method was originally developed to evaluate the oxidative stability of biodiesel-rich fuels, it remains a useful accelerated comparative tool for assessing relative trends in oxidation behavior. Therefore, the induction periods reported here should be interpreted as comparative indicators of oxidative response under standardized accelerated conditions rather than direct predictors of storage lifetime under ambient conditions. The neat fuel (S10 B14 diesel) exhibited an initial IP of 41.77 ± 1.35 h, which decreased to 36.58 ± 0.21 h after 50 days (≈12% reduction) and further to 33.86 ± 0.33 h after 100 days (≈19% reduction). This reduction indicates the progression of oxidative processes during storage, mainly associated with the biodiesel fraction, which contains unsaturated fatty acid esters susceptible to radical-mediated oxidation. The addition of both the commercial additive (CA) and compound **1** resulted in slightly higher IP values at 50 days (37.26 ± 0.70 h and 37.17 ± 0.28 h, respectively) compared to the non-additivated fuel (36.58 ± 0.21 h). Although this represents a modest increase (≈1.6–1.9%), a critical evaluation of the associated standard deviations suggests that these differences may fall within the experimental uncertainty. This overlap between error intervals indicates that the improvement in oxidative stability at this stage is not statistically significant with high confidence. A similar trend is observed after 100 days of storage. The added samples exhibited IP values of 34.89 ± 0.11 h (CA) and 34.33 ± 0.19 h (**1**), compared to 33.86 ± 0.33 h for the neat fuel. While these values indicate a consistent trend toward improved stability in the presence of additives, the magnitude of the enhancement remains small (≈1.4–3.0%), and partial overlap of the error bars persists. Therefore, although both additives appear to retard oxidative degradation, the effect should be interpreted as subtle under the evaluated conditions.

**Figure 4:**
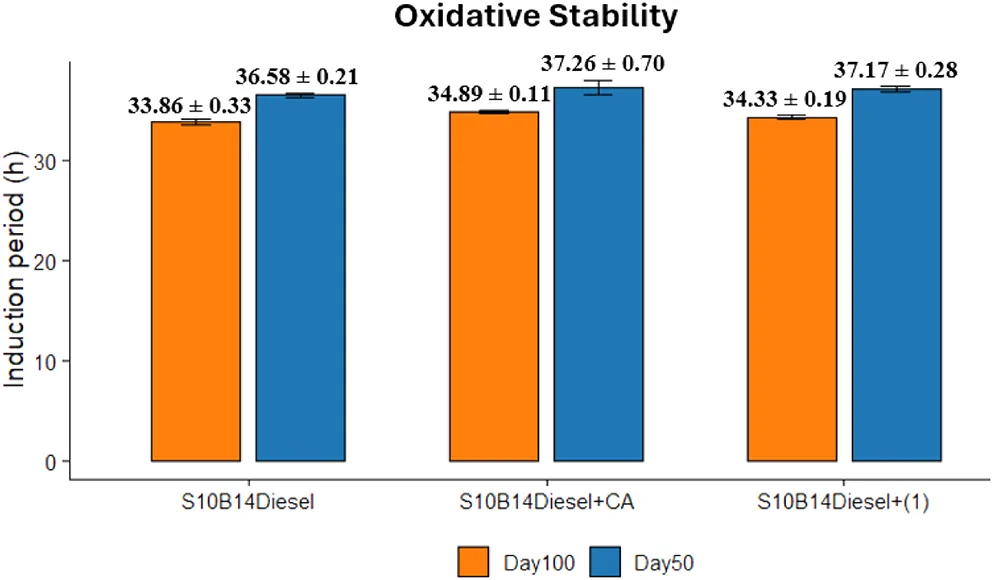
Oxidative stability of diesel-biodiesel blends during storage, expressed as induction period (h), determined by the Rancimat method at 110 °C. Values are reported as mean ± standard deviation (SD), n = 3. Full experimental data, including individual replicate measurements, are provided in Table S14 in the Supplementary Information.

Despite these limitations, compound **1** yielded average IP values that followed the same general trend observed for the commercial additive throughout the storage period. However, considering the associated experimental uncertainties and the overlap of error intervals, the present dataset does not allow statistically significant differences between treatments to be established. Therefore, the observed results should be interpreted with caution and regarded as preliminary evidence only. The relatively small differences between treatments highlight the importance of expanding the experimental design to enable more robust statistical discrimination. Future studies should include longer storage periods, a larger number of replicates, and additional additive concentrations. Furthermore, evaluating different biodiesel blend ratios and complementary oxidation parameters would provide a more comprehensive assessment of fuel degradation and antioxidant performance. Consequently, compound **1** should be regarded as a candidate for further investigation under expanded experimental conditions.

### 3.3 Predicted Oxidative Pathways and Environmental Implications

The reactivity descriptors (Table 1) calculated for **1** show electronic differences compared to those of a similar structure without cyclization **2**. The presence of the fused ring near the aromatic ring of **1** may reduce *π*-conjugation, resulting in different electronic properties compared to **2** (Figure 3c). Under the three conditions evaluated (X-ray geometry, gas-phase geometry, and nonpolar medium geometry), **1** exhibits higher HOMO energies than LUMO and larger *E*_GAP_ values than **2**, indicating greater kinetic stability and lower polarizability. This behavior is observed in the gas-phase (**1**: 573.89 kJ/mol; **2**: 564.90 kJ/mol) as well as in *n*-hexadecane (**1**: 567.24 kJ/mol; **2**: 557.97 kJ/mol) and X-ray geometry (**1**: 563.12 kJ/mol; **2**: 557.74 kJ/mol), suggesting that cyclization rigidifies the electronic system independently of the medium. This characteristic is also observed in the other descriptors. The higher *E*_GAP_ of **1** results in greater chemical hardness (η), which reinforces the lower propensity to undergo electronic disturbances. Compound **2**, with a lower *E*_GAP_, exhibits lower hardness, a characteristic associated with more reactive and electronically flexible systems.

**Table 1:** Chemical Reactivity Descriptors of **1** and **2** ^a^

Descriptor	1 (X-ray geometry)	1 (gas-phase)	1 (*n*-hexadecane)	2 (X-ray geometry)	2 (gas-phase)	2 (*n*-hexadecane)
HOMO Energy (HOMO)	–671.47	–677.64	–685.83	–681.89	–685.20	–692.71
LUMO Energy (LUMO)	–108.35	–103.75	–118.59	–124.15	–120.30	–134.74
Energy gap (*E*_*GAP*_)	563.12	573.89	567.24	557.74	564.90	557.97
Ionization Energy (*I*)	671.47	677.64	685.83	681.89	685.20	692.71
Electronic Affinity (*A*)	108.35	103.75	118.59	124.15	120.30	134.74
Chemical potential (*μ*)	–389.91	–390.69	–402.21	–403.02	–402.75	–413,72
Electronegativity (*χ*)	389.91	390.69	402.21	403.02	402.75	413,72
Chemical hardness (*η*)	281.56	286.96	283.62	278.87	282.45	278.98
Electrophilicity Index (*ω*)	269.97	265.95	285.19	291.22	278,14	306.76

a All values in kJ/mol.

In all phases analyzed, cyclization in **1** results in a less electronegative molecule (χ) and more positive chemical potential values (μ) when compared to the open/chain analog **2**. This property is consistent with its lower *π*-conjugation. The electrophilicity index (ω) is also consistent with this behavior, as a molecule without cyclization exhibits higher electrophilicity values, which suggests a greater capacity to stabilize additional charges and a greater propensity to participate in redox processes. In contrast, **1** exhibits lower electrophilicity, which is consistent with its greater structural rigidity. These descriptors indicate that cyclization does not simply reduce reactivity in a generic sense; rather, it shifts the electronic balance toward a less perturbed and more constrained molecular framework.

The electrophilicity values obtained in *n*-hexadecane show slight variations in comparison with the gas phase values, which would be expected for nonpolar, aromatic systems in solvents with low dielectric constants, as in the case of *n*-hexadecane (ε = 2.0402). However, in the solid-state calculation, a more pronounced stabilization of **1** is observed, possibly associated with the more efficient crystalline packing and additional intermolecular interactions identified in the crystal structure analysis. This observation is important because it links the crystallographic results to the electronic descriptors without overstating their functional consequences. Based on these descriptors, the cyclization present in **1** can impact its electronic properties, reducing conjugation, increasing chemical hardness, and decreasing both electrophilicity and electronegativity.

The MEP map (Figure 3c) shows that regions of negative electrostatic potential (red areas) are concentrated around electronegative atoms, such as oxygen and *π*-systems. In the three phases analyzed, the charge distributions around this group and along the *π*-systems are similar, but the most relevant differences appear near the methoxy substituents on aromatic ring 2 and in the cyclized region adjacent to aromatic ring 1. These differences indicate a redistribution of local electronic environment caused by cyclization, suggesting that cyclization modifies local electronic accessibility without reduce the overall donor–acceptor character of the chalcone skeleton.

The predicted *k*_OH_ values using MACCS descriptors indicate that compounds **1** and chalcone **2** possess a high propensity to react with hydroxyl radicals compared with the main constituents of diesel, biodiesel, and several commercial additives (Table 2). In this context, high *k*_OH_ values suggest that these compounds may efficiently compete with fuel molecules for reactions with ^•^OH radicals, thereby contributing to radical scavenging and potentially limiting the propagation of oxidative chain reactions. However, *k*_OH_ should not be interpreted exclusively as a measure of antioxidant effectiveness or oxidative stability. A high *k*_OH_ value also reflects an increased likelihood that the molecule itself will react with hydroxyl radicals. Therefore, radical-scavenging ability and oxidative persistence must be considered as distinct properties. In the present study, the oxidative persistence of the compounds was further evaluated through reaction simulations and Fenton oxidation experiments, which provide complementary information regarding their structural resistance to oxidative transformation. At the same time, the applicability-domain (AD) values should be considered when interpreting the predicted *k*_OH_ values, particularly for compound **1**, whose AD (65.79%) is lower than that of compound **2** (80.0%) and several reference antioxidants. Therefore, the predicted *k*_OH_ values should be regarded as indicators of relative hydroxyl-radical reactivity rather than as complete descriptors of antioxidant performance. A comprehensive assessment of antioxidant behavior requires consideration of both radical-scavenging reactivity and the resistance of the antioxidant itself to oxidative degradation.

**Table 2:** The Reaction Rate (*k*_OH_) for **1** and **2** ^a^

	Reaction rate coefficient (*k*_OH_) (M^–1^ s^–1^)	
Molecules^b^	AD (%)	MACCS
**1**	65.79	0.90 × 10^10^
**2**	80.0	1.07 × 10^10^
Diesel (C_12_H_23_)	100	6.23 × 10^9^
*n*-hexadecane (C_16_H_34_)	100	6.23 × 10^9^
BD methyl oleate (C_19_H_36_O_2_)	85.0	5.94 × 10^9^
BD methyl linoleate (C_19_H_34_O_2_)	85.0	5.94 × 10^9^
BD methyl palmitate (C_17_H_34_O_2_)	89.47	4.97 × 10^9^
BD methyl stearate (C_19_H_38_O_2_)	89.47	4.97 × 10^9^
TBHQ^86^	100	7.63 × 10^9^
BHT^86^	100	4.34 × 10^9^
BHA^86^	77.78	4.31 × 10^9^
GA^88^	100	1.48 × 10^9^
PG^86^	100	1.22 × 10^10^
PY^86^	100	1.02 × 10^10^

a AD is the % of similarity within the applicability domain.

b In the simulation, we used the compound from this study (**1**) and a similar compound from the literature (**2**).^28^ Diesel and biodiesel (BD) are represented by their major compounds. Diesel is represented by the average molecule (C_12_H_23_) and *n*-hexadecane (C_16_H_34_), which are present and well-representative components of diesel. To represent biodiesel (BD), we used the main components such as methyl oleate (C_19_H_36_O_2_), methyl palmitate (C_17_H_34_O_2_), methyl linoleate (C_19_H_34_O_2_), and methyl stearate (C_19_H_38_O_2_). And we compared them with commercial additives (TBHQ, BHT, BHA, GA, PG, and PY).^86^

The predicted hydroxyl scavenging activity of the compounds was supported by HPLC-MS analysis of the Fenton-incubates of the compounds. Several new m/z peak corresponding to incorporating one or two oxygen atoms into the parent compound could be seen in both cases. High resolution, positive mode HESI MS chromatograms and spectra of the chalcone **2** Fenton-incubate are shown in Figure S3 and Figure S4, respectively. Because of the low peak areas, the structures of the formed derivatives could not be determined from these results.

To better understand the chemical behavior of **1** in a diesel-like medium, reaction simulations were performed using the ASKCOS platform, considering hydroxyl radicals (^•^OH), water (H_2_O), and molecular oxygen (O_2_) as reactive species in C_12_H_23_, an average diesel molecular model. The predicted product distributions, summarized in Figures 5a–c, reveal three distinct mechanistic regimes governed by the electronic structure of **1**. Additional minor products for each condition are provided in Tables S10, S11, and S12. These simulations should be interpreted as exploratory maps of reasonable product space rather than as mechanistic proposal.

**Figure 5:**
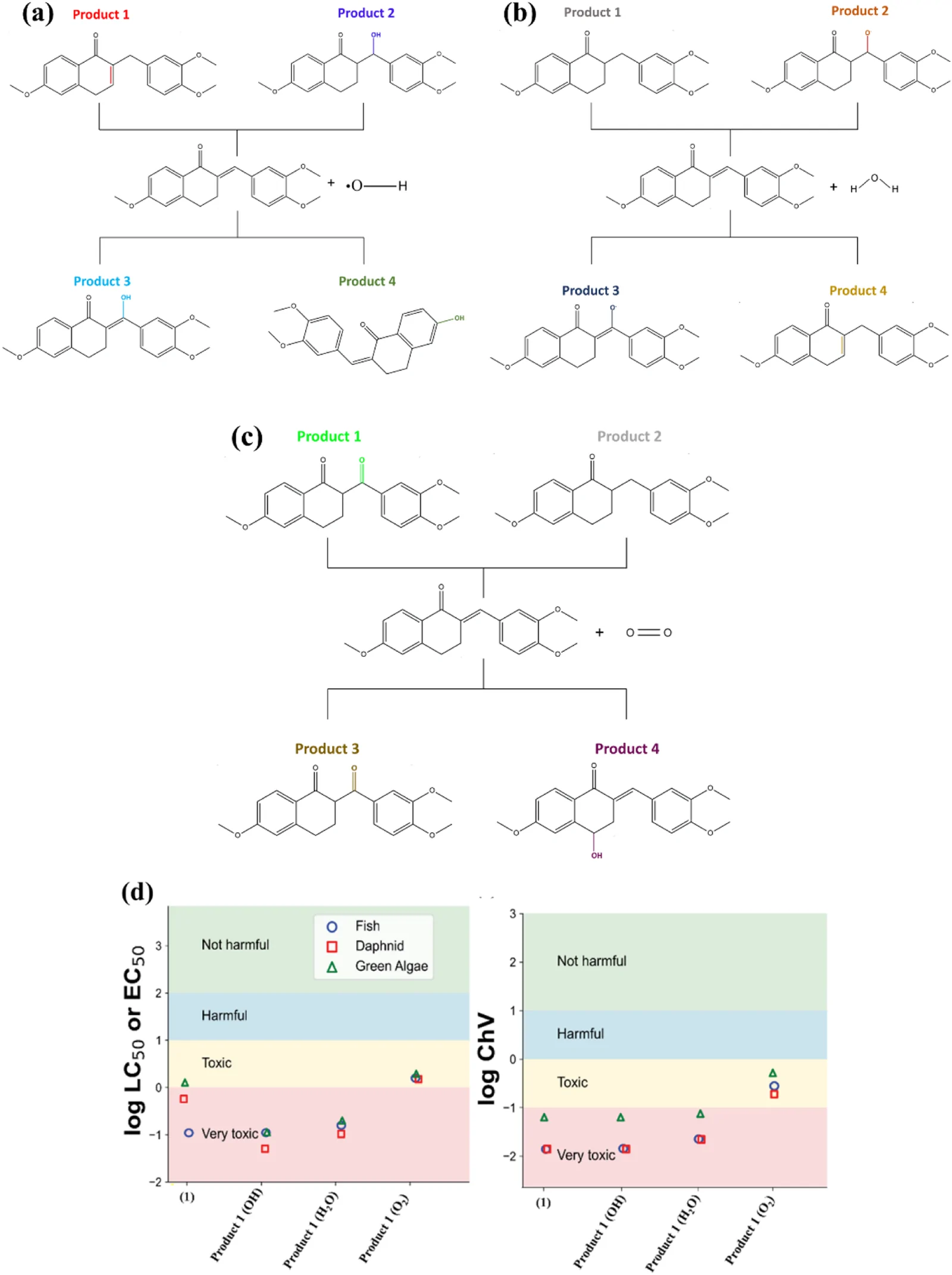
Proposed reaction pathways and major transformation products of compound **1** predicted by ASKCOS in a diesel-like medium (C_12_H_23_ model). (a) Reaction with hydroxyl radicals (^•^OH); (b) reaction with water (H_2_O), **(32c)** reaction with molecular oxygen (O_2_) and (d) toxicity of **1**, obtained with the ECOSAR platform. The toxicity classification is given in Table S13. The toxicity parameters include LC50 (lethal concentration for 50% of the population), EC50 (effective concentration for 50% inhibition of growth or reproduction), and ChV (chronic value, representing concentration causing adverse effects over long-term exposure).

Under ^•^OH-mediated conditions (Figure 5a), the most probable product (84.75%) corresponds to the unchanged structure of **1**, indicating remarkable resistance to permanent structural modification upon radical exposure. The remaining products, each with probabilities below 8%, involve limited hydroxylation or minor rearrangements (Table S10). Hydroxyl radicals are highly electrophilic species that react preferentially with π-systems through radical addition mechanisms.^87^ In *α,β*-unsaturated carbonyl compounds, ^•^OH addition at the β-carbon forms resonance-stabilized β-hydroxyalkyl radicals.^88^ The calculated HOMO distribution of **1** is predominantly delocalized over the conjugated enone and methoxy-substituted aromatic rings (Figure 3c), identifying these regions as electron-rich sites. Because ^•^OH behaves as a strong electrophile, interaction is expected at positions with high HOMO contribution. However, extensive π-delocalization promotes radical stabilization rather than structural fragmentation. Consequently, radical intermediates are electronically buffered, and irreversible oxidation pathways are disfavored, explaining the predominance of the intact molecule. Accordingly, the ASKCOS output is consistent with a picture in which compound **1** can interact with radical species without undergoing extensive irreversible degradation.

According to these and the above results, the experimental reactivity of **1** and **2** with Fenton-reaction-generated hydroxyl radicals^82^ showed differences. HPLC-UV analysis of the Fenton-incubated solutions of the two chalcones showed **2** to be more reactive than **1**. In the 60-minute incubations, the initial HPLC-UV peak area of **2** reduced by 20.8%, while that of **1** only by 9.6%. Computational and experimental findings converge toward the same qualitative interpretation: cyclization appears to reduce the susceptibility of compound **1** to oxidative transformation under hydroxyl-radical conditions. In the ASKCOS simulations, the intact structure remained the most probable outcome under ^•^OH exposure, whereas the Fenton assay showed a smaller reduction in chromatographic peak area for **1** than for **2**. Although these approaches prove different aspects of oxidation chemistry, together they support the view that the cyclic scaffold confers greater resistance to oxidative modification than the open-chain analog.

In contrast, simulations performed with water (Figure 5b) reveal that the most probable products are hydrated derivatives rather than the original structure. The dominant pathway corresponds to 1,4-conjugate (Michael-type) addition of water to the *α,β*-unsaturated system, generating β-hydroxy ketone products. This reactivity pattern is consistent with the LUMO distribution calculated for **1**, which shows significant orbital coefficients at the *β*-carbon and partial contribution at the carbonyl carbon, indicating electrophilic character typical of Michael acceptors. In polar reactions, regioselectivity is governed by LUMO topology rather than HOMO density. The moderate HOMO–LUMO gap suggests sufficient stabilization of the conjugated framework, while still allowing nucleophilic addition under favorable conditions. The products depicted in Figure 5b represent the most probable hydration pathways, while less favored rearrangements and secondary products are summarized in Table S11. Unlike the radical scenario, the transformation under aqueous conditions reflects polar addition rather than radical stabilization.

A third mechanistic profile emerges in the presence of molecular oxygen (Figure 5c). Ground-state O_2_ is a triplet diradical and typically reacts with carbon-centered radicals, forming peroxyl intermediates in autoxidation processes.^89^ In this case, the most probable products correspond to oxygen insertion and subsequent oxidation at activated positions within the conjugated system, rather than retention of the original structure. The products shown in Figure 5c reflect likely peroxyl-derived or oxygenated intermediates, whereas additional minor oxidation products are compiled in Table S12. The susceptibility of specific positions to oxygen incorporation can be rationalized by considering spin and electron-density redistribution after initial activation of the *π*-system. The HOMO delocalization facilitates transient radical formation, and once a localized radical is generated, O_2_ trapping becomes kinetically feasible. Nevertheless, extensive conjugation still limits uncontrolled autoxidative chain propagation, indicating a balance between activation and stabilization.

To further assess the environmental implications of the predicted transformation pathways, an ecotoxicological evaluation was performed using the ECOSAR predictive model. The results are summarized in Figure 5d, which presents the acute toxicity (log LC_50_ or EC_50_) and chronic toxicity (log ChV) values for the compound **1** and its major transformation products derived from the ^•^OH, H_2_O, and O_2_ reactions. The complete numerical dataset, including acute (LC_50_, EC_50_) and chronic (ChV) toxicity values for fish, daphnia, and green algae, as well as log K_ow_ values, is provided in Table S15. Toxicity classification was interpreted according to the Toxicity Classification Criteria established by the European Union (EU CLP Regulation) and corresponding Chinese environmental hazard guidelines. Under these frameworks, substances are categorized as “very toxic”, “toxic”, “harmful”, or “not harmful” based on threshold LC_50_/EC_50_ values expressed in mg L^–1^. In Figure 5d, the colored regions represent these regulatory categories, allowing direct visualization of environmental hazard levels.

For the original molecule **1**, the predicted acute toxicity values fall predominantly within the “toxic” to “very toxic” range for aquatic organisms, particularly for daphnia and algae, indicating significant baseline ecotoxicological concern. The chronic toxicity (ChV) values reinforce this classification, with several endpoints positioned within the “very toxic” region. This behavior is consistent with the relatively high hydrophobicity of **1**, as reflected by its log K_ow_ value of 4.682. The log K_ow_ is a key physicochemical descriptor that serves as a proxy for lipophilicity, bioaccumulation potential, and membrane permeability. Compounds with log K_ow_ values between 3 and 5 are generally considered highly hydrophobic and susceptible to bioaccumulation in aquatic organisms.^90^ The **1** exhibits a log K_ow_ of 4.682, indicating strong lipophilicity and potential for bioaccumulation.

The major transformation products exhibit log K_ow_ values of 4.682, 4.451, and 3.093, respectively. The hydroxyl radical-derived major product maintains a log K_ow_ essentially identical to the parent compound (4.682), consistent with the minimal structural alteration predicted in Figure 5a. This finding explains the similar toxicity profile observed in Figure 5d, where acute and chronic toxicity values remain within comparable hazard categories. In contrast, the hydration product (Figure 5b) shows a reduced log K_ow_ of 4.451, indicating slightly increased polarity due to hydroxyl incorporation. Although this reduction marginally lowers bioaccumulation potential, the compound remains within the hydrophobic regime associated with aquatic toxicity. Accordingly, ECOSAR predicts moderate shifts in LC_50_/EC_50_ values, but not a dramatic decrease in hazard classification. More pronounced changes are observed for the oxygen-derived oxidation product (Figure 5c), which presents a log K_ow_ of 3.093. This substantial reduction reflects increased polarity and reduced lipophilicity following oxygen insertion. Therefore, predicted acute and chronic toxicity values shift toward less severe categories, particularly for fish and algae. The lower hydrophobicity likely reduces membrane partitioning and internal exposure, thereby decreasing the ecotoxicological impact.

These results reveal an important structure–toxicity relationship. While the parent compound **1** demonstrates strong radical-stabilizing capacity and limited deep oxidative degradation under ^•^OH exposure, its hydrophobic character inherently contributes to aquatic toxicity. However, oxidative transformation-especially oxygen incorporation-tends to increase molecular polarity and reduce log K_ow_, partially mitigating ecotoxicological risk. From an application standpoint, future studies should aim to balance oxidation resistance with reduced ecotoxicological impact, either by structural modification of the chalcone skeleton or by evaluation of lower effective concentrations in fuel formulations. Although compound **1** demonstrated greater resistance toward oxidative transformation than compound **2**, the ECOSAR predictions reveal an important limitation regarding its environmental profile. The predicted log K_ow_ value (4.682) together with the aquatic toxicity classifications suggest that increased molecular persistence may be accompanied by enhanced environmental persistence and bioaccumulation potential. This highlights an inherent trade-off frequently encountered during the design of antioxidant additives, where structural features that improve oxidative resistance may also reduce environmental compatibility. Therefore, antioxidant performance alone should not be considered sufficient for selecting candidate fuel additives. These findings indicate that future optimization of the chalcone scaffold should not focus exclusively on oxidative stabilization, but also on reducing hydrophobicity and predicted aquatic toxicity. Possible design strategies include the incorporation of more polar substituents, modulation of lipophilicity, or introduction of structural features that favor environmental degradation while preserving the electronic characteristics associated with radical scavenging. Such strategies may improve the balance between antioxidant performance and environmental safety.

## 4 Final Considerations

The structural, electronic, predictive, and experimental results collectively indicate that the cyclic chalcone analog **1** exhibits enhanced rigidity and distinct oxidative-response behavior when compared to its non-cyclized analog **2**. Single-crystal X-ray diffraction confirmed that cyclization near aromatic ring 1 induces conformational restriction and influences molecular packing, leading to a more constrained supramolecular arrangement stabilized predominantly by C–H···O and C–H···π interactions. Hirshfeld surface analysis revealed that these weak interactions account for approximately 95% of the intermolecular contacts, contributing to the structural stabilization of the crystal lattice. DFT calculations showed that **1** presents consistently higher HOMO-LUMO energy gaps across solid-state, gas-phase, and nonpolar medium conditions, indicating greater kinetic stability and lower electronic flexibility relative to the open-chain analog. The increased chemical hardness (η), reduced electrophilicity (ω), and lower electronegativity (χ) confirm that cyclization decreases *π*-conjugation while reinforcing structural robustness. Despite its greater rigidity, compound **1** exhibited substantial predicted reactivity toward hydroxyl radicals (*k*_OH_ = 0.90 × 10^10^ M^–1^ s^–1^), indicating a strong ability to compete with fuel constituents for reactions with ^•^OH radicals. However, this parameter alone does not establish the oxidative stability of the additive itself. Reaction simulations using the ASKCOS platform revealed that, under radical conditions in a diesel-like medium, the most probable reaction outcome (84.75%) corresponds to preservation of the original structure of **1**, while the Fenton assay showed that **1** undergoes a smaller decrease in chromatographic peak area than (**2**) after hydroxyl-radical exposure. These observations support the interpretation that the cyclic scaffold is less susceptible to oxidative transformation. Rancimat experiments performed on S10 B14 diesel showed that samples containing compound **1** exhibited slightly higher induction-period values than the untreated fuel, consistent with the greater oxidative persistence observed in the complementary mechanistic analyses. Although the stabilization effect was modest under the evaluated conditions and should be interpreted cautiously considering the experimental variability, the results indicate that cyclization influences the oxidative-response behavior of chalcones in fuel media. Because the experimental methodologies adopted in this work were designed primarily to provide structural, mechanistic, and comparative information, these findings should not be interpreted as direct validation of practical fuel performance under commercial storage conditions.

An equally important outcome of this study concerns the environmental profile of the investigated scaffold. ECOSAR predictions indicate considerable aquatic toxicity together with relatively high hydrophobicity for compound **1** and its major transformation products, demonstrating that resistance to oxidative degradation alone cannot be considered a sufficient criterion for selecting future fuel additives. Consequently, the principal contribution of this work lies in elucidating how cyclization simultaneously affects molecular structure, electronic properties, oxidative-response behavior, and environmental profile. These findings provide a rational framework for the future design of chalcone-based fuel additives in which oxidative stabilization and environmental compatibility are optimized simultaneously.

## Supplementary Material



## Notes

Crystallographic and structural data; FMO and MEP analyses; HPLC-MS and HESI-MS data; molecular structures (SMILES); ASKCOS reaction simulations; toxicity and ecotoxicological data; Rancimat oxidative stability data; statistical analysis code (PDF)
