# Supplementary material for: Cyclization-Modulated Structure and Oxidative Behavior of Methoxy-Substituted Chalcones: Implications for Diesel-Biodiesel Additive Design
Source: ACS Omega. 2026 Sep 2;11(36):54359–72. doi: 10.1021/acsomega.6c05813 (PMC13584782; doi:10.1021/acsomega.6c05813)
Supplement: Supplementary file 1 [file ao6c05813_si_001.pdf]

Cyclization-Modulated Structure and Oxidative Behavior of Methoxy-Substituted Chalcones: Implications for Diesel-Biodiesel Additive Design

Renata Layse G. de Paula[a], Vitor S. Duarte[a], Pollyana P. Firmino[c], Javier Ellena[c], Gábor Bognár[d], Pál Perjési[d], Júlio César de O. Ribeiro[e], Valter H Carvalho-Silva[a], Allen G. Oliver[b] and Hamilton B. Napolitano[a,b*]

[a] Center for BioMolecular Stability of the Cerrado, State University of Goiás, Anápolis, GO, Brazil.
[b] Department of Chemistry and Biochemistry, University of Notre Dame, Notre Dame, IN, USA.
[c] Sao Carlos Institute of Physics, University of Sao Paulo, Sao Carlos, SP, Brazil.
[d] Institute of Pharmaceutical Chemistry, University of Pécs, Pécs, Hungary.
[e] Institute of Chemistry, Federal University of Goiás, Goiânia, GO, Brazil.

*hbnapolitano@gmail.com

Supplementary Information

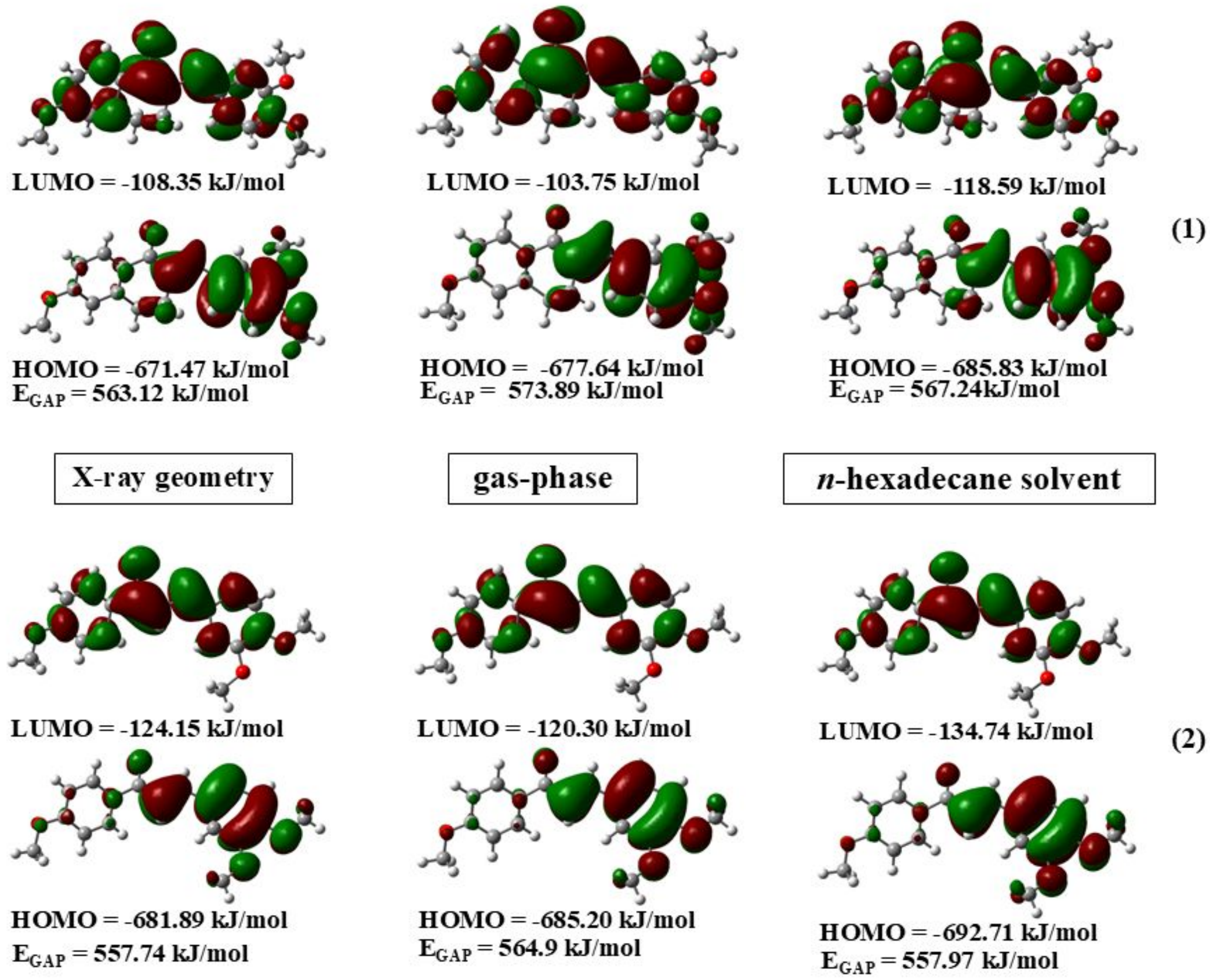

Figure S1. The FMO orbitals for **(1)** and **(2)** in the X-ray geometry, gas-phase, and *n*-hexadecane.

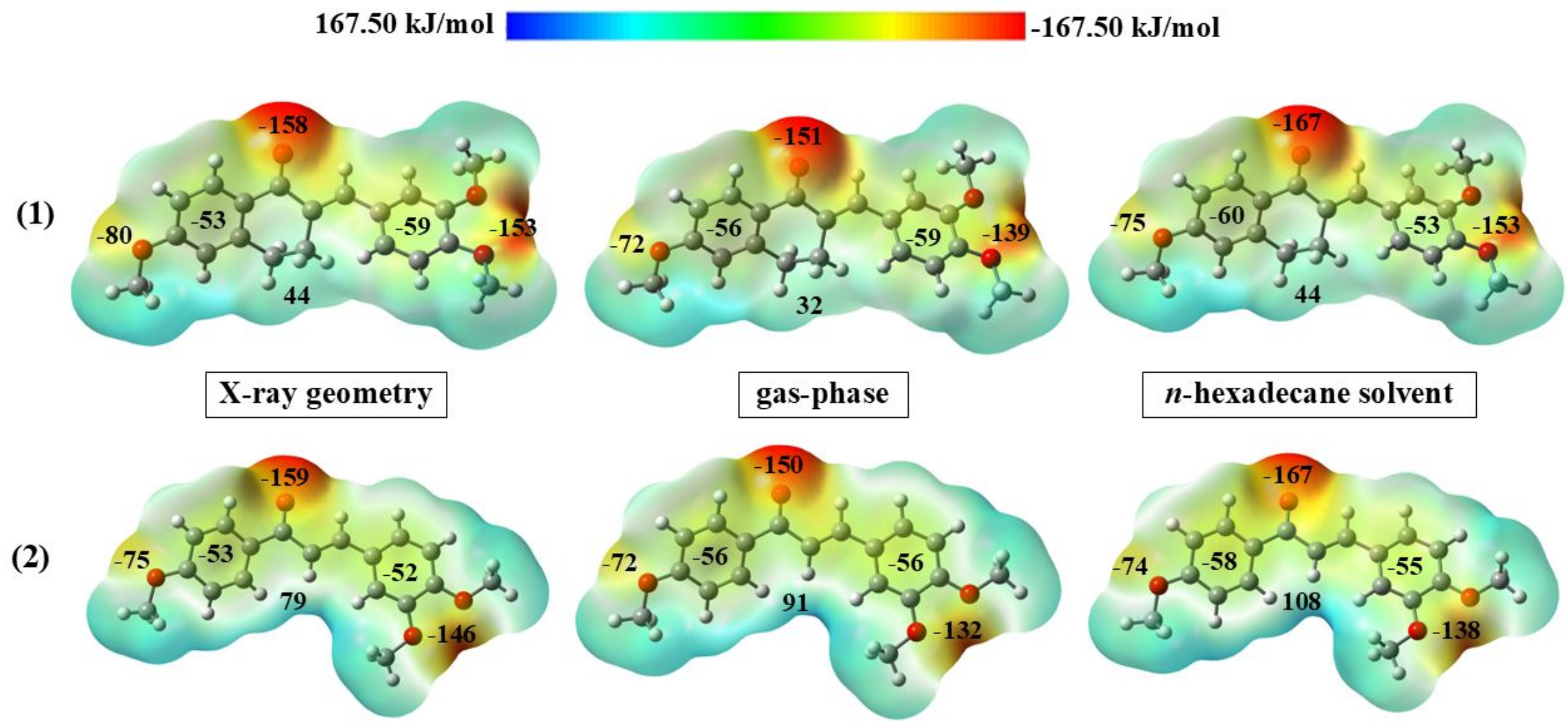

Figure S2. The MEP map surfaces for **(1)** and **(2)** in the X-ray geometry, gas-phase, and *n*-hexadecane environments.

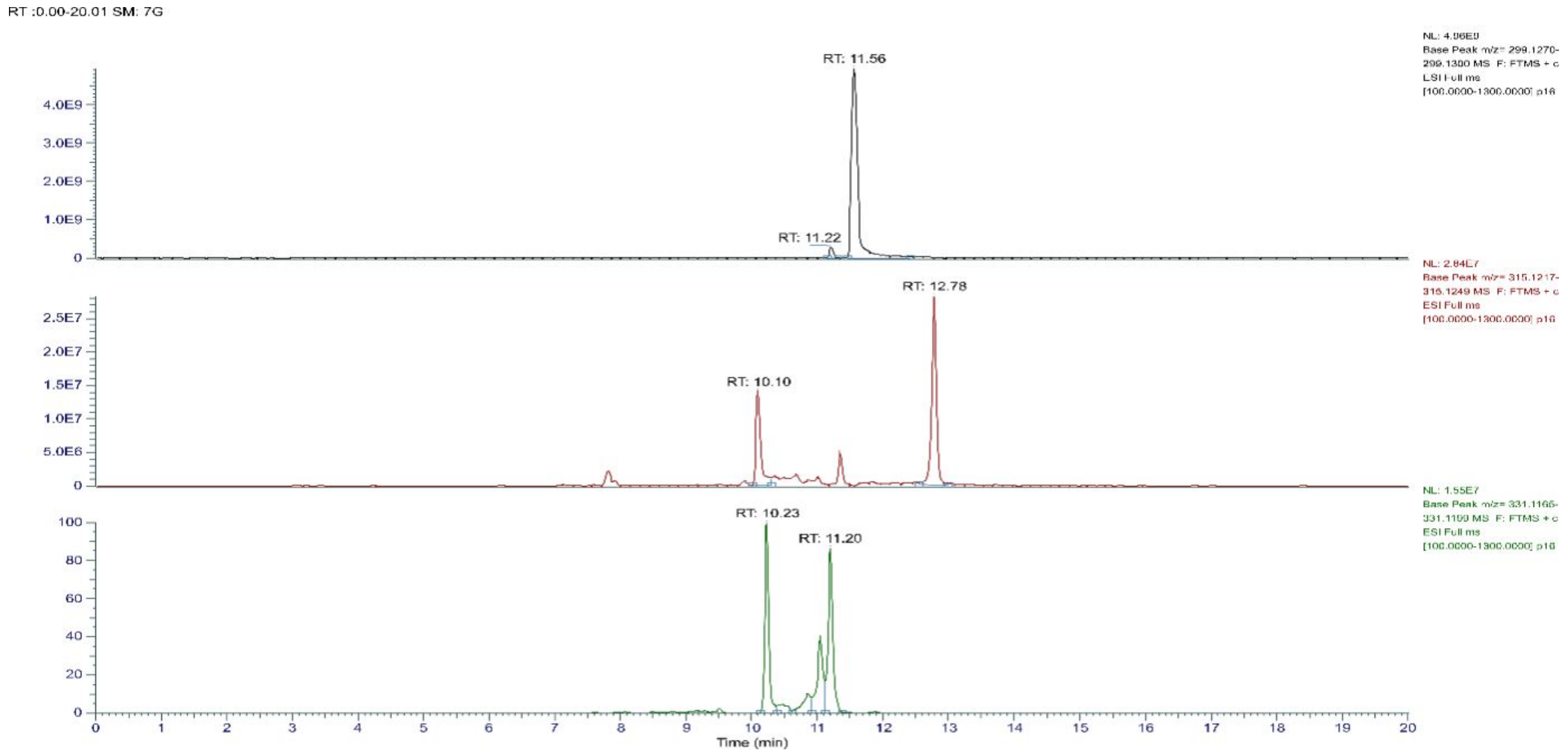

Figure S3. High resolution, positive mode HPLC-MS chromatograms of the chalcone (**2**) Fenton-incubate.

Panel I.: HPLC-MS extracted ion chromatogram (m/z 299.1283 $[(\mathbf{2})+H]^{+}$) of the chalcone (**2**) (Z-isomer tr 11.22 min and E-isomer tr 11.56 min).

Panel II.: HPLC-MS extracted ion chromatogram (m/z 315.1233 $[(\mathbf{2})+\mathbf{O}+H]^{+}$) of the chalcone (**2**)**+"O"** (tr 10.10 min and tr 12.78 min).

Panel III.: HPLC-MS extracted ion chromatogram (m/z 331.1182 $[(\mathbf{2})+\mathbf{2O}+H]^{+}$) of the chalcone (**2**)**+2"O"** (tr 10.23 min and tr 11.20 min).

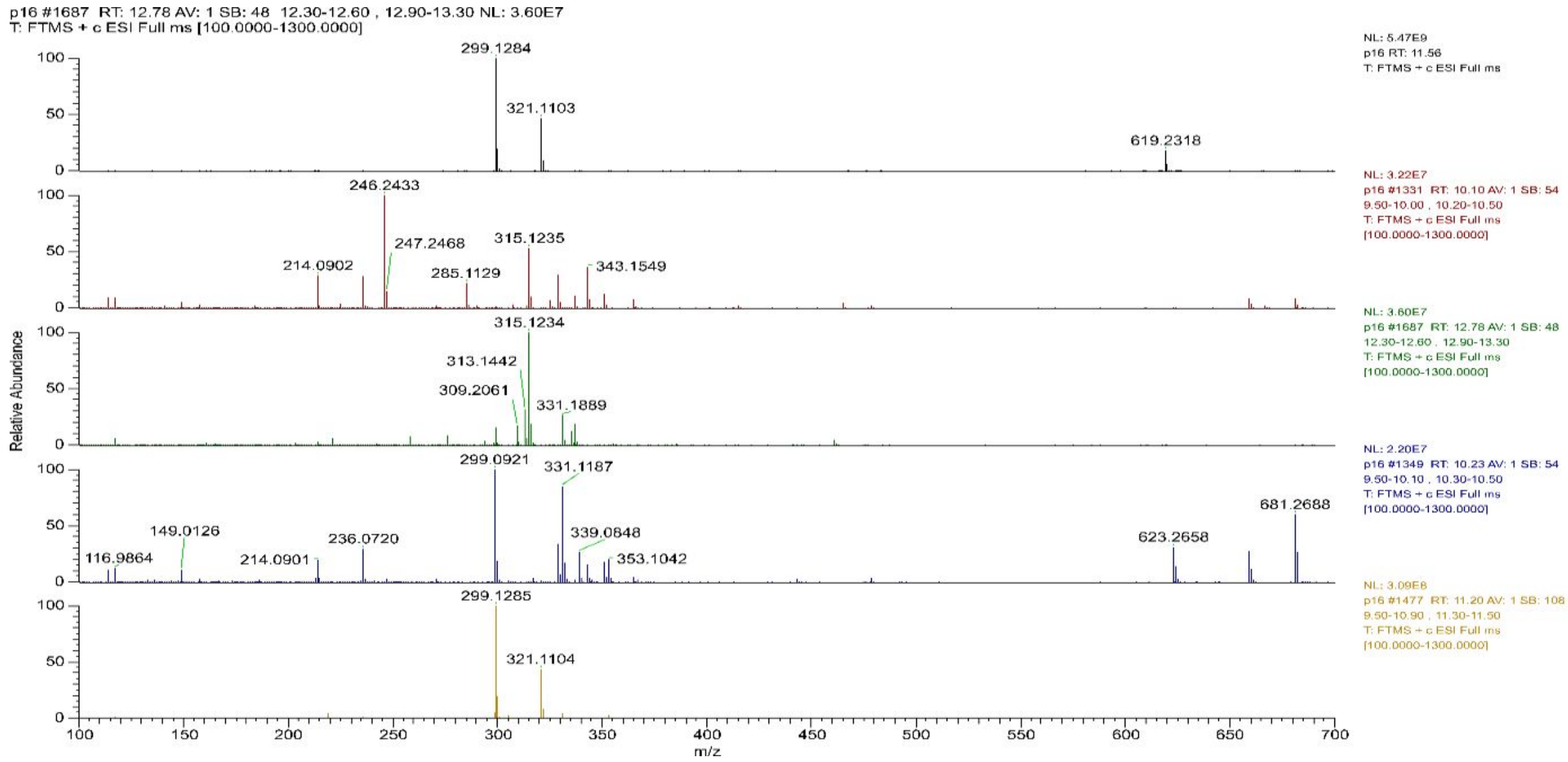

Figure S4. High resolution, positive mode HESI MS spectra of the chalcone (**2**) Fenton-incubate.

Panel I.: High resolution, positive mode HESI MS spectrum of the E-isomer of the chalcone (**2**) (m/z 299.1284 $[(\mathbf{2})+H]^+$, m/z 321.1103 $[(\mathbf{2})+Na]^+$, dimer m/z 619.2318 $[(\mathbf{2})_2+Na]^+$), tr 11.56 min.

Panel II.: High resolution, positive mode HESI MS spectrum of the chalcone (**2**)+"**O**" (m/z 315.1235 $[(\mathbf{2})+\mathbf{O}+H]^+$), tr 10.10 min. *Note: peak intensity low, background higher!*

Panel III.: High resolution, positive mode HESI MS spectrum of the chalcone (**2**)+"**O**" (m/z 315.1234 $[(\mathbf{2})+\mathbf{O}+H]^+$), tr 12.78 min. *Note: peak intensity low, background higher!*

Panel IV.: High resolution, positive mode HESI MS spectrum of the chalcone (**2**)+"**2O**" (m/z 331.1187 $[(\mathbf{2})+2\mathbf{O}+H]^+$), tr 10.23 min. *Note: peak intensity low, background higher!*

Panel V.: High resolution, positive mode HESI MS spectrum of the Z-isomer of the chalcone (**2**) and the chalcone (**2**)+"**2O**" (*note: coeluting peaks, intensity of Z-isomer is very high, (2)+"**2O**" intensity very low, m/z is not visible*); (m/z 299.1285 $[(\mathbf{2})+H]^+$, m/z 321.1104 $[(\mathbf{2})+Na]^+$), tr 11.20 min.

Table S1. Crystallographic data and structure refinement parameters for compound **(1)**.

Formula	$C_{20}H_{20}O_4$
Formula weight	324,376 g/mol
Temperature	100.0(2) K
Wavelength	1.54184 Å
Crystal system, space group, Z	Monoclinic, $P2_1/c$, 4
Unit cell dimensions	a = 15.2682(3) Å<br>b = 15.0511(3) Å<br>c = 7.0468(1) Å<br>α = 90°<br>β = 94.363(2)°<br>γ = 90°
Volume	1614.68(5) $Å^3$
Calculated density	1.334 g/cm^3
Absorption coefficient	0.752 mm^{-1}
Goodness-of-fit (S)	1.0466
Final R índices [$I > 2\sigma(I)$]	R_1 = 0.0455; wR_2 = 0.1204
R indices (all data)	R_1 = 0.0521; wR_2 = 0.1255

Table S2. Main supramolecular interactions observed in the compound **(1)**.

**Interactions**	**d(D–H)Å**	**d(H···A)Å**	**d(D–A)Å**	**(D–H–A)(°)**	**Symmetry code**
C20–H20b···O2	0.990	2.562(5)	3.482(5)	154.69(4)	1-x,1-y,-z
C1–H1b···O3	0.980	2.661(4)	3.453(5)	138.20(6)	1-x,1-y,1-z
C17–H17b···O4	0.980	2.430(4)	3.407(5)	174.58(6)	-x,1-y,1-z
C17–H17c···Cg1	0.980	3.719(5)	4.439(6)	132.28(5)	1-x,1-y,-z
C3–H3···Cg1	0.950	3.162(5)	3.772(4)	123.61(6)	x,1/2-y,-1/2+z
C15–H15···Cg2	0.950	3.292(6)	4.129(4)	148.14(6)	x,1/2-y,-1/2+z

Table S3. Fractional Atomic Coordinates (×10[4]) and Equivalent Isotropic Displacement Parameters (Å[2]×10[3]) for **(1)**. U_{eq} is defined as 1/3 of the trace of the orthogonalised U_{ij}.

**Atom**	**x**	**y**	**z**	U_{eq}
O3	975.0(7)	4776.1(7)	4254.4(15)	31.7(3)
O1	8800.6(7)	3338.5(7)	747.4(15)	34.0(3)
O4	1284.7(7)	3806.8(7)	7218.3(15)	32.2(3)
O2	4721.1(7)	3701.0(9)	-1459.4(15)	38.5(3)
C13	3174.9(9)	3912.7(9)	3238(2)	25.6(3)
C10	6578.2(9)	3978.1(9)	1969.1(19)	25.0(3)
C7	4667.0(9)	3858.1(9)	1907.1(19)	25.3(3)
C5	6099.5(9)	3713.0(9)	297.0(19)	25.6(3)
C11	7489.9(9)	3889.8(9)	2139(2)	26.4(3)
C12	3786.2(10)	3936.3(9)	1725(2)	26.2(3)
C2	7917.7(9)	3509.5(9)	657(2)	27.4(3)
C17	2721.5(9)	3357.0(9)	6245(2)	26.8(3)
C14	2376.4(9)	4385.6(9)	2992(2)	26.2(3)
C8	5233.7(9)	3838.0(9)	3752.6(19)	26.0(3)
C18	3331.7(9)	3399.0(9)	4873(2)	26.2(3)
C16	1939.4(9)	3821.4(9)	5998(2)	26.5(3)
C6	5127.1(10)	3770.5(9)	103(2)	27.4(3)
C4	6545.4(10)	3362.9(9)	-1208(2)	28.6(3)
C15	1766.9(9)	4348.0(9)	4352(2)	26.7(3)
C9	6091.9(9)	4346.0(9)	3577(2)	28.0(3)
C3	7442.8(10)	3255.3(9)	-1026(2)	30.2(3)
C20	1374.1(10)	3173.8(11)	8731(2)	34.2(3)
C19	789.9(10)	5368.7(11)	2694(2)	37.1(4)
C1	9304.9(10)	3557.3(12)	2478(2)	39.0(4)

Table S4. Anisotropic Displacement Parameters (×10^4) **(1)**. The anisotropic displacement factor exponent takes the form: $-2p^2[h^2a^{*2} \times U_{11} + ... +2hka^* \times b^* \times U_{12}]$.

**Atom**	U_{11}	U_{22}	U_{33}	U_{23}	U_{13}	U_{12}
O3	26.1(5)	33.5(5)	35.3(5)	10.5(4)	-0.1(4)	3.9(4)
O1	30.1(5)	37.9(6)	34.4(6)	0.0(4)	5.0(4)	3.2(4)
O4	29.3(5)	34.5(6)	32.7(5)	8.4(4)	2.5(4)	4.0(4)
O2	34.4(6)	55.5(7)	24.7(5)	0.5(5)	-4.1(4)	-3.2(5)
C13	26.8(7)	21.3(6)	27.9(7)	-3.0(5)	-3.1(5)	-2.7(5)
C10	30.6(7)	19.7(6)	24.7(6)	1.0(5)	1.0(5)	-0.2(5)
C7	29.6(7)	20.9(6)	24.8(7)	-0.8(5)	-1.1(5)	-0.4(5)
C5	31.1(7)	21.3(6)	24.0(7)	2.6(5)	0.9(5)	-1.1(5)
C11	29.6(7)	24.7(6)	24.7(6)	0.7(5)	-0.2(5)	-0.8(5)
C12	30.6(7)	21.6(6)	25.4(7)	0.1(5)	-4.2(5)	-1.5(5)
C2	28.6(7)	23.3(6)	30.6(7)	2.5(5)	4.2(5)	-0.3(5)
C17	30.6(7)	21.8(6)	26.9(7)	1.5(5)	-4.3(5)	-0.7(5)
C14	27.1(7)	22.4(6)	28.1(7)	2.7(5)	-4.9(5)	-2.9(5)
C8	26.4(7)	27.0(7)	24.3(6)	-3.0(5)	-0.5(5)	1.2(5)
C18	25.2(6)	21.4(6)	31.0(7)	-2.5(5)	-4.2(5)	-0.1(5)
C16	27.6(7)	23.6(6)	27.8(7)	-0.4(5)	-1.1(5)	-2.2(5)
C6	33.3(7)	23.6(6)	24.7(7)	1.2(5)	-1.7(5)	-2.1(5)
C4	36.6(8)	25.5(7)	23.7(7)	-0.6(5)	1.7(6)	-6.0(5)
C15	25.0(7)	23.2(6)	31.0(7)	0.9(5)	-3.9(5)	-0.8(5)
C9	27.4(7)	29.0(7)	27.0(7)	-5.9(5)	-1.1(5)	0.6(5)
C3	38.2(8)	24.8(7)	28.8(7)	-2.3(5)	9.7(6)	-3.6(6)
C20	37.4(8)	35.6(8)	29.5(7)	7.1(6)	2.1(6)	1.0(6)
C19	28.1(7)	39.5(8)	43.3(8)	16.8(7)	1.0(6)	5.8(6)
C1	26.2(7)	49.2(9)	41.6(9)	-0.4(7)	1.7(6)	0.2(7)

Table S5. Bond Lengths in Å for **(1)**.

Atom	Atom	Length/Å
O3	C15	1.3671(17)
O3	C19	1.4274(18)
O1	C2	1.3690(18)
O1	C1	1.430(2)
O4	C16	1.3676(17)
O4	C20	1.4284(18)
O2	C6	1.2261(18)
C13	C18	1.393(2)
C13	C14	1.411(2)
C13	C12	1.4699(19)
C10	C11	1.394(2)
C10	C5	1.397(2)
C10	C9	1.5067(18)

Atom	Atom	Length/Å
C7	C12	1.346(2)
C7	C6	1.5043(19)
C7	C8	1.5066(19)
C5	C4	1.4057(19)
C5	C6	1.483(2)
C11	C2	1.3960(19)
C2	C3	1.396(2)
C17	C16	1.383(2)
C17	C18	1.394(2)
C14	C15	1.387(2)
C8	C9	1.5302(19)
C16	C15	1.413(2)
C4	C3	1.376(2)

Table S6. Bond Angles in ° for **(1)**.

**Atom**	**Atom**	**Atom**	**Angle/°**
C15	O3	C19	117.12(11)
C2	O1	C1	117.43(11)
C16	O4	C20	116.56(11)
C18	C13	C14	118.30(13)
C18	C13	C12	122.33(13)
C14	C13	C12	119.28(12)
C11	C10	C5	120.01(12)
C11	C10	C9	121.09(12)
C5	C10	C9	118.90(12)
C12	C7	C6	117.03(12)
C12	C7	C8	126.01(12)
C6	C7	C8	116.95(12)
C10	C5	C4	119.42(13)
C10	C5	C6	121.13(12)
C4	C5	C6	119.42(13)
C10	C11	C2	119.72(13)
C7	C12	C13	127.84(13)
O1	C2	C3	115.72(12)

**Atom**	**Atom**	**Atom**	**Angle/°**
O1	C2	C11	123.87(13)
C3	C2	C11	120.38(13)
C16	C17	C18	120.39(13)
C15	C14	C13	120.89(13)
C7	C8	C9	111.06(11)
C13	C18	C17	121.11(13)
O4	C16	C17	125.30(13)
O4	C16	C15	115.19(12)
C17	C16	C15	119.49(13)
O2	C6	C5	120.79(13)
O2	C6	C7	121.98(13)
C5	C6	C7	117.11(12)
C3	C4	C5	120.66(13)
O3	C15	C14	125.68(12)
O3	C15	C16	114.51(12)
C14	C15	C16	119.81(13)
C10	C9	C8	110.55(11)
C4	C3	C2	119.74(12)

Table S7. Torsion Angles in ° for **(1)**.

**Atom**	**Atom**	**Atom**	**Atom**	**Angle/°**
C11	C10	C5	C4	-0.4(2)
C9	C10	C5	C4	-179.55(12)
C11	C10	C5	C6	177.87(12)
C9	C10	C5	C6	-1.32(19)
C5	C10	C11	C2	-2.1(2)
C9	C10	C11	C2	177.11(12)
C6	C7	C12	C13	171.23(13)
C8	C7	C12	C13	-7.9(2)
C18	C13	C12	C7	-32.5(2)
C14	C13	C12	C7	151.14(14)
C1	O1	C2	C3	-177.61(13)
C1	O1	C2	C11	0.7(2)
C10	C11	C2	O1	-175.23(12)
C10	C11	C2	C3	3.0(2)
C18	C13	C14	C15	0.7(2)
C12	C13	C14	C15	177.21(12)
C12	C7	C8	C9	-141.73(13)
C6	C7	C8	C9	39.15(16)
C14	C13	C18	C17	-0.6(2)
C12	C13	C18	C17	-177.00(12)
C16	C17	C18	C13	0.6(2)
C20	O4	C16	C17	-8.4(2)
C20	O4	C16	C15	170.24(13)
C18	C17	C16	O4	177.89(12)
C18	C17	C16	C15	-0.7(2)
C10	C5	C6	O2	166.03(13)
C4	C5	C6	O2	-15.7(2)
C10	C5	C6	C7	-17.75(18)
C4	C5	C6	C7	160.47(12)
C12	C7	C6	O2	-5.7(2)
C8	C7	C6	O2	173.49(13)
C12	C7	C6	C5	178.12(12)
C8	C7	C6	C5	-2.68(17)
C10	C5	C4	C3	1.9(2)
C6	C5	C4	C3	-176.33(12)
C19	O3	C15	C14	-5.3(2)
C19	O3	C15	C16	175.61(13)
C13	C14	C15	O3	-179.80(13)
C13	C14	C15	C16	-0.8(2)
O4	C16	C15	O3	1.18(18)
C17	C16	C15	O3	179.92(12)
O4	C16	C15	C14	-177.94(12)
C17	C16	C15	C14	0.8(2)
C11	C10	C9	C8	-140.59(13)
C5	C10	C9	C8	38.59(17)
C7	C8	C9	C10	-56.33(15)
C5	C4	C3	C2	-1.0(2)
O1	C2	C3	C4	176.93(12)
C11	C2	C3	C4	-1.4(2)

Table S8. Hydrogen Fractional Atomic Coordinates (×10^4) and Equivalent Isotropic Displacement Parameters (Å^2×10^3) for **(1)**. U_{eq} is defined as 1/3 of the trace of the orthogonalised U_{ij}.

**Atom**	**x**	**y**	**z**	U_{eq}
H11	7818.49	4087.57	3258.54	32
H12	3528.62	4016.44	466.58	31
H17	2843.48	3007.42	7356.27	32
H14	2254.66	4734.48	1879.72	31
H8A	5368.1	3213.75	4109.24	31
H8B	4909.59	4107.18	4772.82	31
H18	3863.49	3071.72	5056.87	31
H4	6223.93	3198.8	-2362.15	34
H9A	5961.43	4982.62	3342.53	34
H9B	6465.38	4297.39	4784.28	34
H3	7738.02	3009.03	-2042.65	36
H20A	1448.67	2578.69	8202.58	51
H20B	846.46	3185.15	9440.11	51
H20C	1888.67	3323.41	9588.03	51
H19A	798.05	5039.56	1495.32	56
H19B	1235.69	5838.12	2731.23	56
H19C	208.91	5635.27	2783.33	56
H1A	9925.77	3425.86	2343.9	59
H1B	9097.66	3205.1	3524.39	59
H1C	9236.73	4190.92	2751.23	59

Table S9. Smiles for compounds in the study.

**Compound**	**Smile**
(**1**)	COc1cc(ccc1OC)\C=C1/CCc2cc(ccc2C1=O)OC
(**2**)	COc1cc(ccc1OC)\C=C\C(=O)c1ccc(cc1)OC
Diesel ($C_{12}H_{23}$)	CCCCCCCCCCCC
n-hexadecane ($C_{16}H_{34}$)	CCCCCCCCCCCCCCCC
Methyl Palmitate ($C_{17}H_{34}O_2$) – C16:0 (C16, saturated)	CCCCCCCCCCCCCCCC(=O)OC
Methyl Stearate ($C_{19}H_{38}O_2$) – C18:0 (C18, saturated)	CCCCCCCCCCCCCCCCCC(=O)OC
Methyl Oleate ($C_{19}H_{36}O_2$) – C18:1 (C18, unsaturated, 1 cis double bond)	CCCCCCCCC=CCCCCCCCC(=O)OC
Methyl Linoleate ($C_{19}H_{34}O_2$) – C18:2 (2 double bonds)	CCCCCC=CCC=CCCCCCCC(=O)OC

Table S10. Reaction simulation using the ASKCOS platform for: Reactants: **(1)**; Agents: •OH; Solvent: $C_{12}H_{23}$ (average diesel molecule).

Rank	Product	Smile	Probability
1	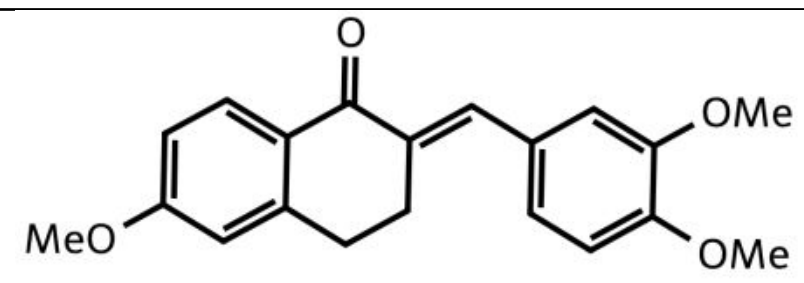	COc1ccc2c(c1)CC/C(=C\c1ccc(OC)c(OC)c1)C2=O	0.8475
2	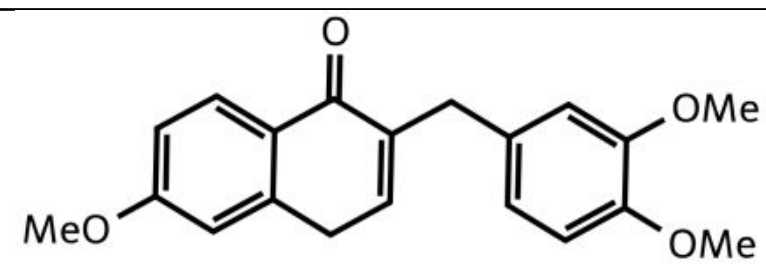	COc1ccc2c(c1)CC=C(Cc1ccc(OC)c(OC)c1)C2=O	0.0795
3	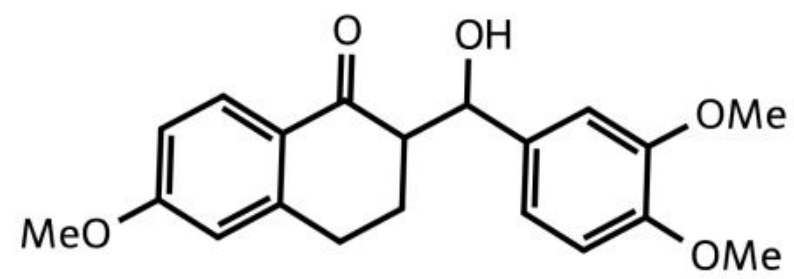	COc1ccc2c(c1)CCC(C(O)c1ccc(OC)c(OC)c1)C2=O	0.0356
4	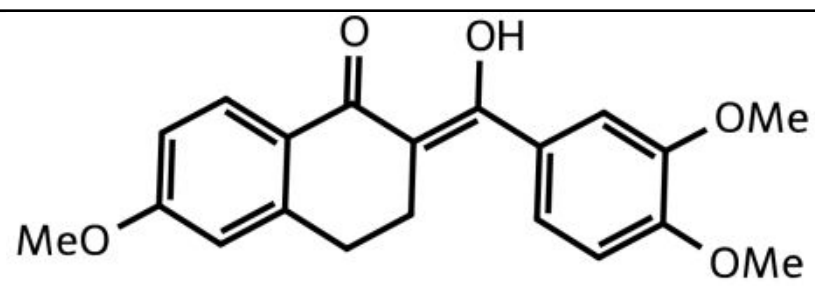	COc1ccc2c(c1)CCC(=C(O)c1ccc(OC)c(OC)c1)C2=O	0.0085
5	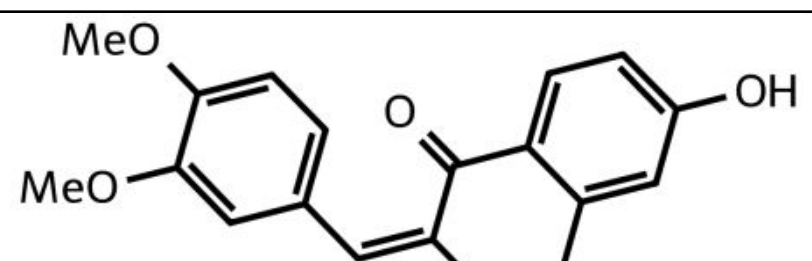	COc1ccc(C=C2CCc3cc(O)ccc3C2=O)cc1OC	0.0079
6	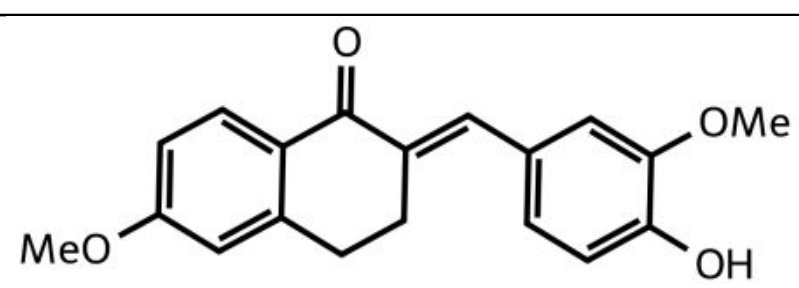	COc1ccc2c(c1)CCC(=Cc1ccc(O)c(OC)c1)C2=O	0.0065
7	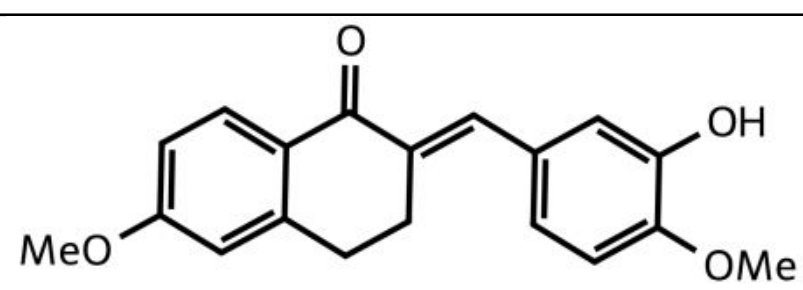	COc1ccc2c(c1)CCC(=Cc1ccc(OC)c(O)c1)C2=O	0.0050
8	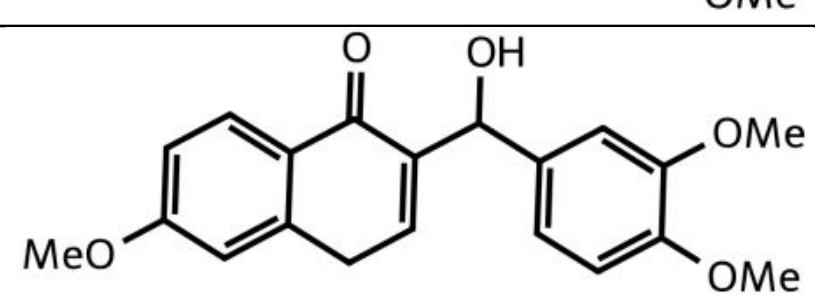	COc1ccc2c(c1)CC=C(C(O)c1ccc(OC)c(OC)c1)C2=O	0.0047
9	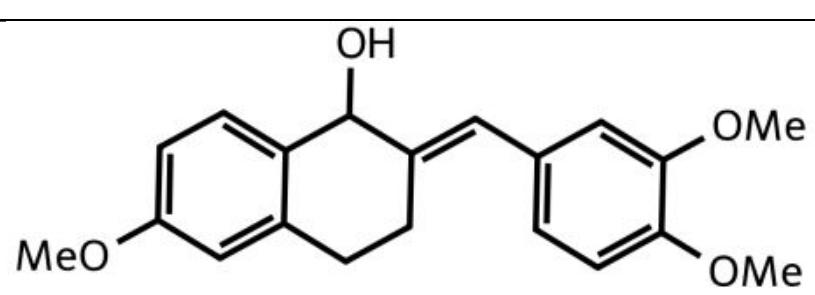	COc1ccc2c(c1)CCC(=Cc1ccc(OC)c(OC)c1)C2O	0.0015
10	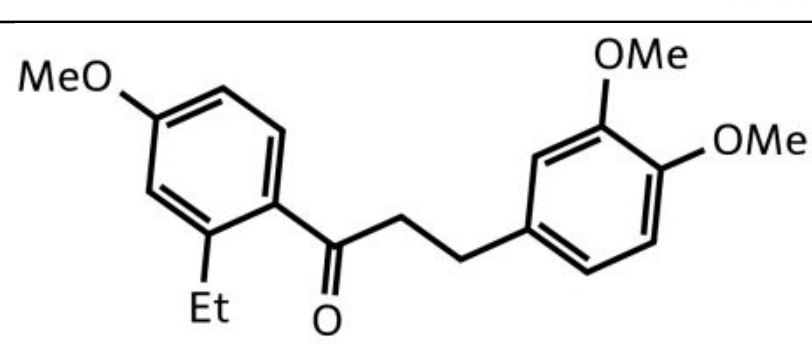	CCc1cc(OC)ccc1C(=O)CCc1ccc(OC)c(OC)c1	0.0011

Table S11. Reaction simulation using the ASKCOS platform for: Reactants: **(1)**; Agents: H_2O; Solvent: $C_{12}H_{23}$ (average diesel molecule).

Rank	Product	Smile	Probability
1	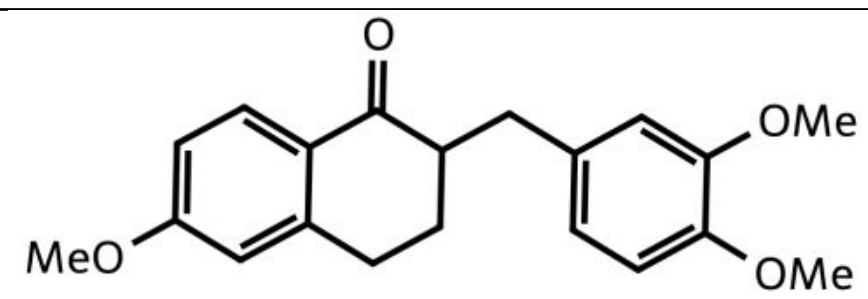	COc1ccc2c(c1)CCC(Cc1ccc(OC)c(OC)c1)C2=O	0.5285
2	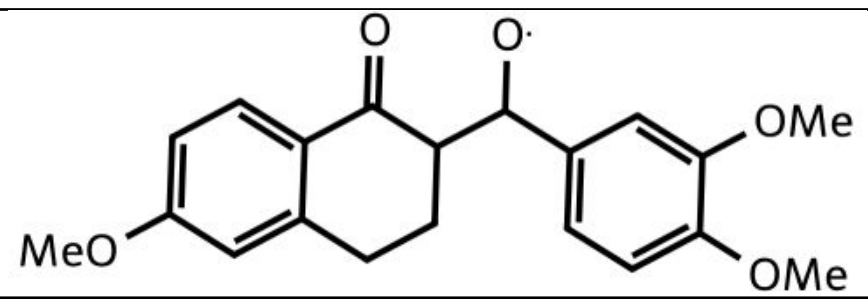	COc1ccc2c(c1)CCC(C([O])c1ccc(OC)c(OC)c1)C2=O	0.2924
3	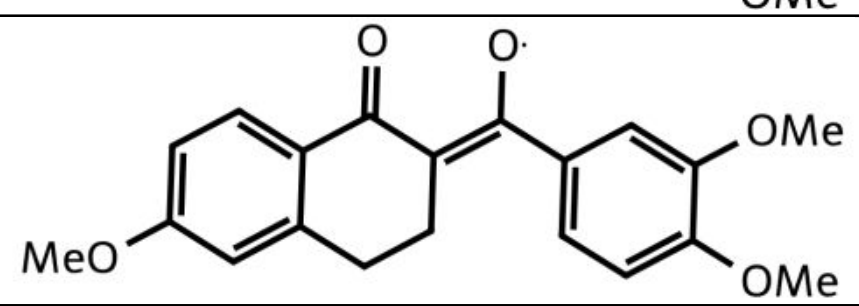	COc1ccc2c(c1)CCC(=C([O])c1ccc(OC)c(OC)c1)C2=O	0.1023
4	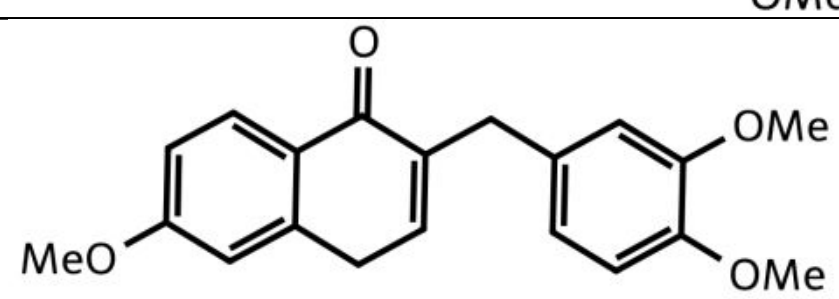	COc1ccc2c(c1)CC=C(Cc1ccc(OC)c(OC)c1)C2=O	0.0496
5	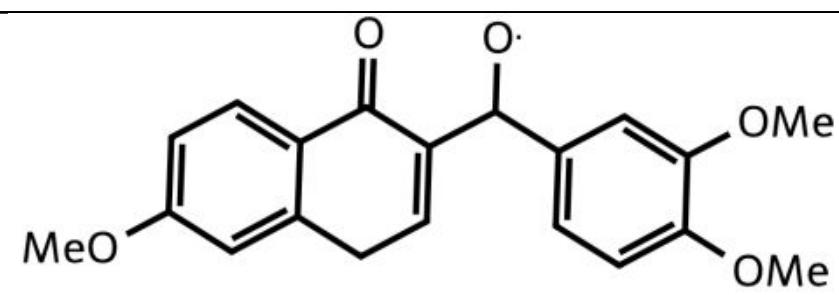	COc1ccc2c(c1)CC=C(C([O])c1ccc(OC)c(OC)c1)C2=O	0.0113
6	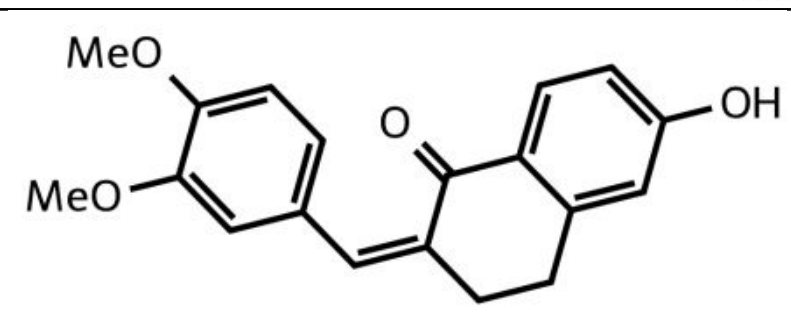	COc1ccc(C=C2CCc3cc(O)ccc3C2=O)cc1OC	0.0049
7	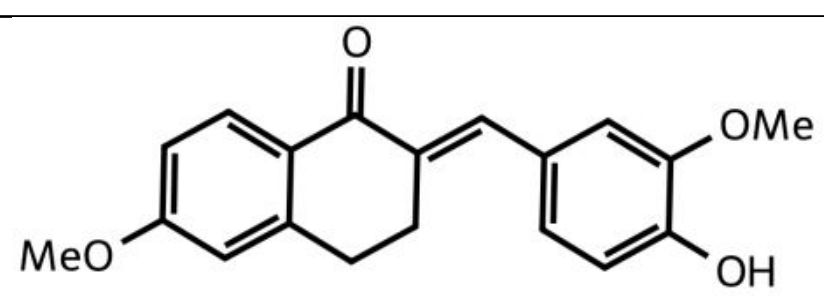	COc1ccc2c(c1)CCC(=Cc1ccc(O)c(OC)c1)C2=O	0.0041
8	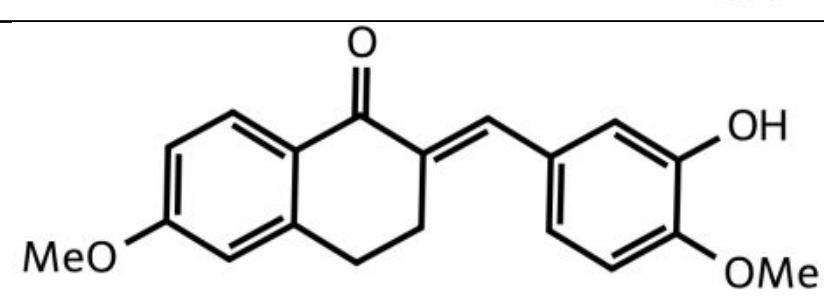	COc1ccc2c(c1)CCC(=Cc1ccc(OC)c(O)c1)C2=O	0.0031
9	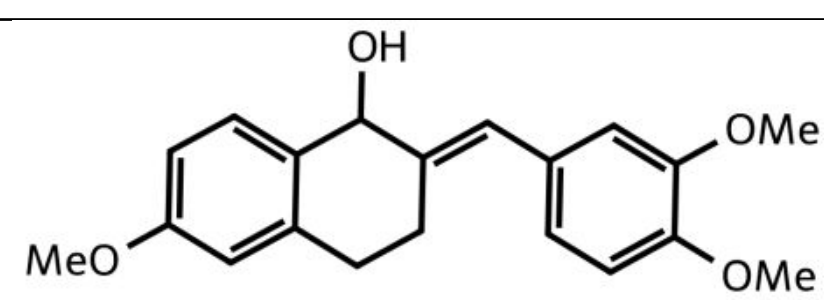	COc1ccc2c(c1)CCC(=Cc1ccc(OC)c(OC)c1)C2O	0.0010
10	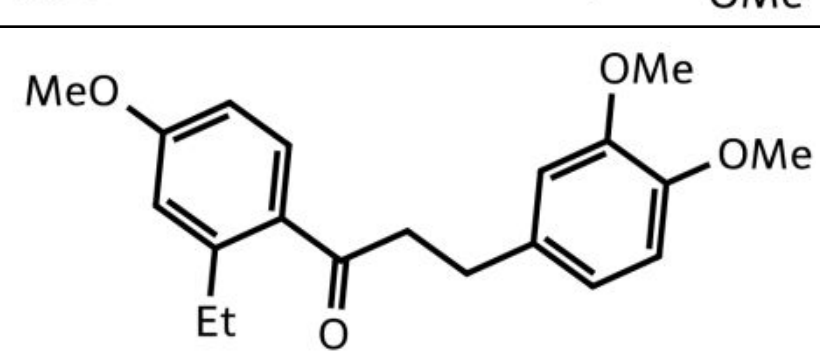	CCc1cc(OC)ccc1C(=O)CCc1ccc(OC)c(OC)c1	0.0007

Table S12. Reaction simulation using the ASKCOS platform for: Reactants: **(1)**; Agents: O_2; Solvent: $C_{12}H_{23}$ (average diesel molecule).

Rank	Product	Smile	Probability
1	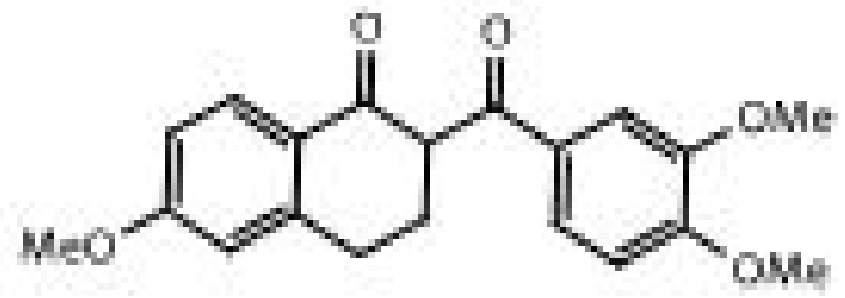	COc1ccc2c(c1)CCC(C(=O)c1ccc(OC)c(OC)c1)C2=O	0.4543
2	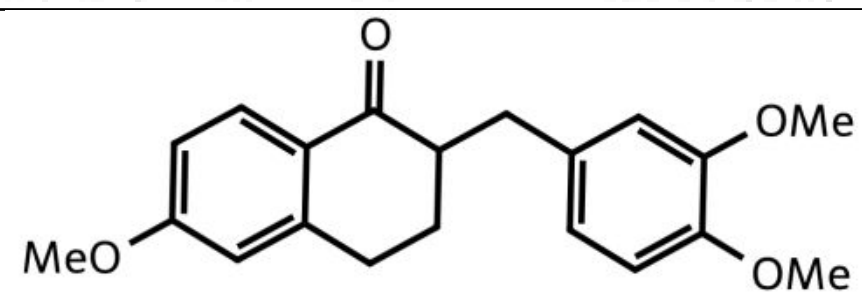	COc1ccc2c(c1)CCC(Cc1ccc(OC)c(OC)c1)C2=O	0.1532
3	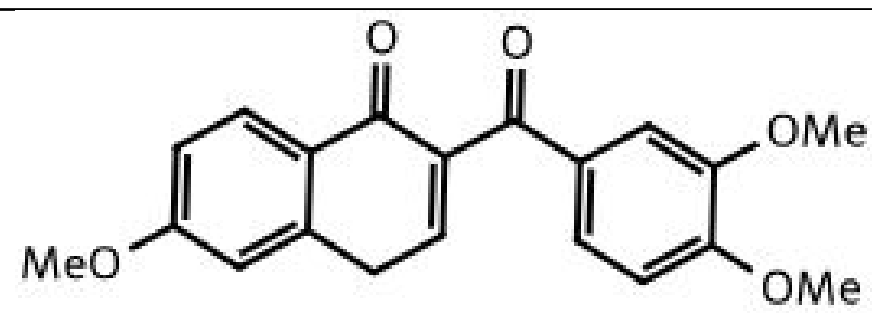	COc1ccc2c(c1)CC=C(C(=O)c1ccc(OC)c(OC)c1)C2=O	0.1091
4	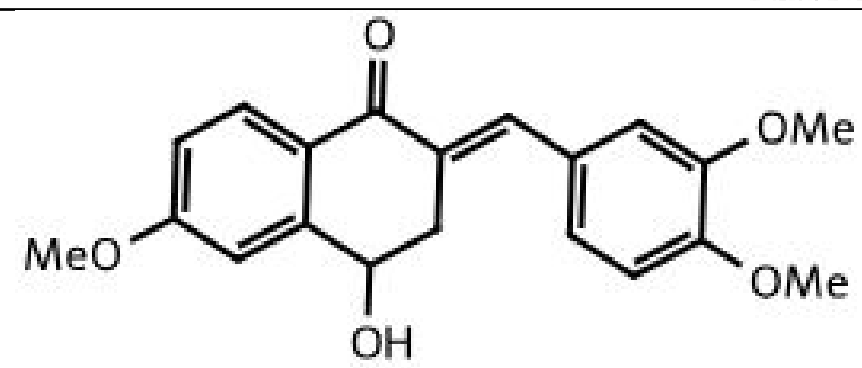	COc1ccc2c(c1)C(O)CC(=Cc1ccc(OC)c(OC)c1)C2=O	0.1021
5	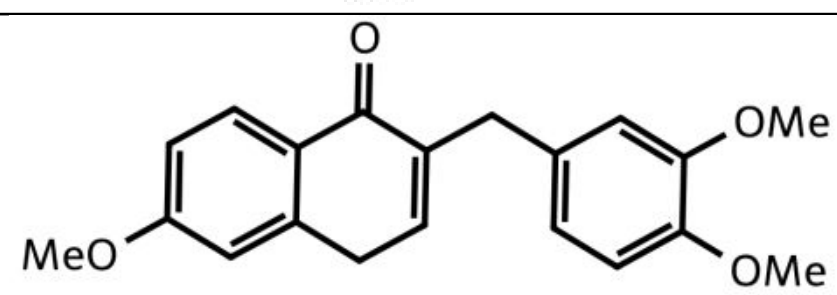	COc1ccc2c(c1)CC=C(Cc1ccc(OC)c(OC)c1)C2=O	0.0692
6	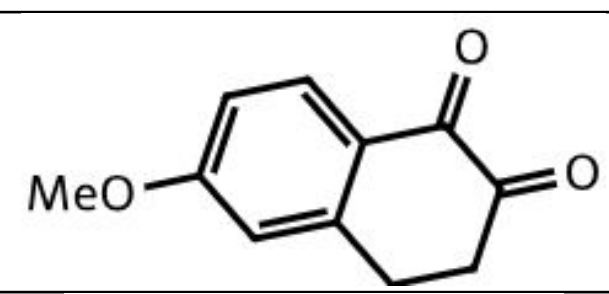	COc1ccc2c(c1)CCC(=O)C2=O	0.0439
7	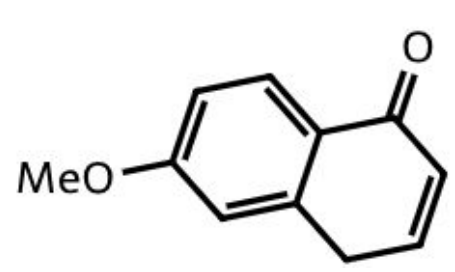	COc1ccc2c(c1)CC=CC2=O	0.0205
8	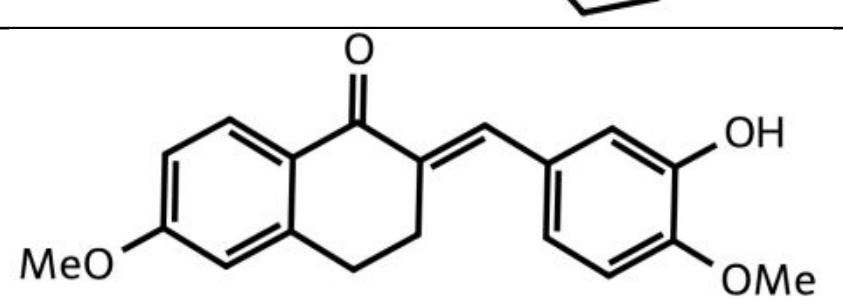	COc1ccc2c(c1)C(O)CC(C(=O)c1ccc(OC)c(OC)c1)C2=O	0.0145
9	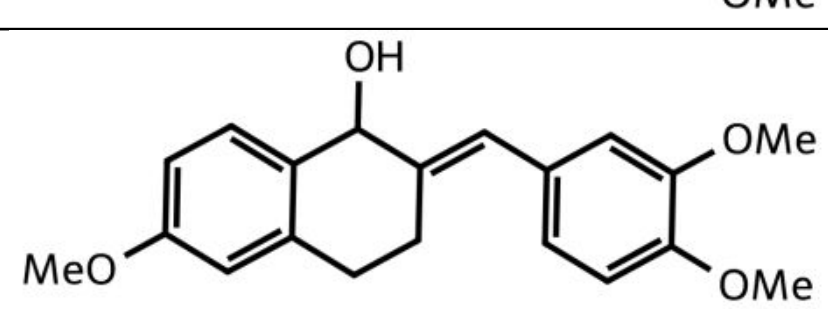	COc1ccc2c(c1)CCCC2=O	0.0056
10	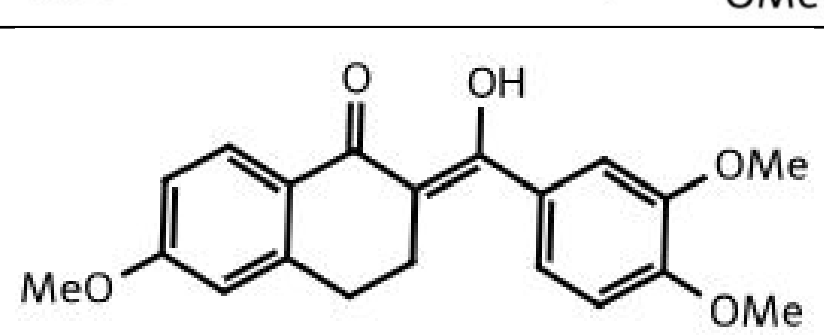	COc1ccc2c(c1)CCC(=C(O)c1ccc(OC)c(OC)c1)C2=O	0.0045

Table S13. Toxicity classification criteria according to European Union and Chinese guidelines. The table summarizes acute and chronic toxicity thresholds (LC_{50}, EC_{50}, ChV) used for categorizing chemicals as "not harmful," "harmful," "toxic," or "very toxic," based on Directive 67/548/EEC (EU) and HJ/T 154–2004 (China).

Acute toxicity [1]	Chronic toxicity [2]	Toxicity grade
$LC_{50} > 100$ or $EC_{50} > 100$	ChV > 10	Not harmful
$10 < LC_{50} < 100$ or $10 < EC_{50} < 100$	1 < ChV < 10	Harmful
$1 < LC_{50} < 10$ or $1 < EC_{50} < 10$	0.1 < ChV < 1	Toxic
$LC_{50} < 1$ or $EC_{50} < 1$	ChV < 0.1	Very toxic

[1] Criteria set by the European Union (described in Annex VI of Directive 67/548/EEC).

[2] Criteria set by the Chinese Hazard Evaluation Guidelines for New Chemical Substances (HJ/T 154–2004).

Table S14a. Rancimat data for diesel-biodiesel blends during storage (0, 50, and 100 days), expressed as induction period (h), including triplicate measurements and average values.

	**Day 0**				**Day 50**				**Day 100**			
	**T1 IP (h)**	**T2 IP (h)**	**T3 IP (h)**	**Average (h)**	**T1 IP (h)**	**T2 IP (h)**	**T3 IP (h)**	**Average (h)**	**T1 IP (h)**	**T2 IP (h)**	**T3 IP (h)**	**Average (h)**
**S10 B14 diesel**	42.19	42.88	40.26	**41.77**	36.35	36.77	36.62	**36.58**	33.98	34.13	33.49	**33.86**
**S10 B14 diesel + CA***	-	-	-	-	37.58	36.45	37.75	**37.26**	35.02	34.82	34.83	**34.89**
**S10 B14 diesel + (1)**	-	-	-	-	36.88	37.20	37.45	**37.17**	34.11	34.44	34.44	**34.33**

* CA: commercial additive. Measurements were performed in triplicate (T1–T3), and average values are reported.

Table S14b. Oxidative stability of diesel-biodiesel blends during storage, expressed as induction period (h), measured by the Rancimat method at 110 °C

**Sample**	**Day 0**	**Day 50**	**Day 100**
**S10 B14 diesel**	41.77 ± 1.35	36.58 ± 0.21	33.86 ± 0.33
**S10 B14 diesel + CA***	-	37.26 ± 0.70	34.89 ± 0.11
**S10 B14 diesel + (1)**	-	37.17 ± 0.28	34.33 ± 0.19

Values expressed as mean ± **standard deviation** (SD), n = 3

Table S14c. Oxidative stability of diesel-biodiesel blends during storage, expressed as induction period (h), measured by the Rancimat method at 110 °C

**Sample**	**Day 0**	**Day 50**	**Day 100**
**S10 B14 diesel**	41.77 ± 0.78	36.58 ± 0.12	33.86 ± 0.19
**S10 B14 diesel + CA***	-	37.26 ± 0.40	34.89 ± 0.06
**S10 B14 diesel + (1)**	-	37.17 ± 0.16	34.33 ± 0.11

Values are expressed as mean ± **standard error** of the mean (SE), n = 3

Table S15. Ecotoxicological parameters for **(1)** and major transformation products of reaction with •OH, H_2O and O_2Values include acute (LC_{50}, EC_{50}) and chronic (ChV) toxicity to fish, daphnia and algae, as well log $K_{ow,}$ and water solubility.

Toxicity	**(1)**	Prod1- •OH	Prod1- H_2O	Prod1- O_2
LC_{50} (fish 96 h)	1.040	1.04	1.69	19.20
LC_{50} (daphnia 48 h)	0.755	0.755	1.20	18.30
EC_{50} (green algae 96 h)	1.570	1.570	2.28	20.70
ChV (fish, chronic)	0.136	0.136	0.215	3.210
ChV (daphnia, chronic)	0.147	0.147	0.220	2.370
ChV (green algae, chronic)	0.712	0.712	0.989	6.790
Log K_{ow}	4.682	4.682	4.451	3.093
Water solubility (mg L^{-1})	0.884	0.884	1.357	16.16

Statistical analysis code for SD and SEM calculations

```
#Manuscript_Unveiling Oxidative Stability of Cyclic Chalcones as Proxy on Diesel-Biodiesel Additives

library(dplyr)
library(ggplot2)
library(writexl)

data <- data.frame(
  day = c(rep("Day0", 3),
        rep("Day50", 9),
        rep("Day100", 9)),

  samples = c(rep("S10B14Diesel", 3),
            rep(c("S10B14Diesel", "S10B14Diesel+CA", "S10B14Diesel+(1)"), each = 3),
            rep(c("S10B14Diesel", "S10B14Diesel+CA", "S10B14Diesel+(1)"), each = 3)),

  values = c(
    42.19, 42.88, 40.26,
    36.35, 36.77, 36.62,
    37.58, 36.45, 37.75,
    36.88, 37.2, 37.45,
    33.98, 34.13, 33.49,
    35.02, 34.82, 34.83,
    34.11, 34.44, 34.44
  )
)

summary <- data %>%
```

```
  group_by(day, samples) %>%
  summarise(
    mean = mean(values),
    standard_desviation = sd(values),
    standard_error = sd(values) / sqrt(n()),
    n = n(),
    .groups = "drop"
  )

print(summary)

final_result <- merge(data, summary, by = c("day", "samples"))
write.csv(final_result, "full_result.csv", row.names = FALSE)



data$samples <- factor(
  data$samples,
  levels = c(
    "S10B14Diesel",
    "S10B14Diesel+CA",
    "S10B14Diesel+(1)"
  )
)

summary$samples <- factor(
  summary$samples,
  levels = c(
    "S10B14Diesel",
    "S10B14Diesel+CA",
```

```
    "S10B14Diesel+(1)"
  )
)
summary_plot <- summary %>%
  filter(day %in% c("Day50", "Day100"))

grafic_sd <- ggplot(summary_plot,
                    aes(x = samples, y = mean, fill = day)) +
  geom_col(
    position = position_dodge(0.7),
    width = 0.6,
    color = "black"
  ) +
  geom_errorbar(
    aes(
      ymin = mean - standard_desviation,
      ymax = mean + standard_desviation
    ),
    position = position_dodge(0.7),
    width = 0.2
  ) +
  labs(
    title = "Oxidative Stability (SD)",
    x = "",
    y = "Induction period (h)"
  ) +
  scale_fill_manual(values = c(
    "Day50" = "#1f77b4",
    "Day100" = "#ff7f0e"
```

```
  )) +
  theme_classic(base_size = 14) +
  theme(
    plot.title = element_text(hjust = 0.5, face = "bold"),
    legend.position = "bottom"
  )

grafic_sd

grafic_se <- ggplot(summary_plot,
                    aes(x = samples, y = mean, fill = day)) +
  geom_col(
    position = position_dodge(0.7),
    width = 0.6,
    color = "black"
  ) +
  geom_errorbar(
    aes(
      ymin = mean - standard_error,
      ymax = mean + standard_error
    ),
    position = position_dodge(0.7),
    width = 0.2
  ) +
  labs(
    title = "Oxidative Stability (SE)",
    x = "",
    y = "Induction period (h)"
  ) +
```

```
  scale_fill_manual(values = c(
    "Day50" = "#1f77b4",
    "Day100" = "#ff7f0e"
  )) +
  theme_classic(base_size = 14) +
  theme(
    plot.title = element_text(hjust = 0.5, face = "bold"),
    legend.position = "bottom"
  )

grafic_se
```
